# Barriers and facilitators to the uptake of new medicines into clinical practice: a systematic review

**DOI:** 10.1186/s12913-021-07196-4

**Published:** 2021-11-05

**Authors:** Kristina Medlinskiene, Justine Tomlinson, Iuri Marques, Sue Richardson, Katherine Stirling, Duncan Petty

**Affiliations:** 1 Medicine Optimisation Research Group, School of Pharmacy and Medical Sciences, University of Bradford, Richmond Road, Bradford, BD7 1DP UK; 2 Medicine Management and Pharmacy Services, Leeds Teaching Hospitals NHS Trust, Leeds, UK; 3 Department of Management, Huddersfield Business School, University of Huddersfield, Huddersfield, HD1 3DH UK

**Keywords:** New medicines, Uptake, Implementation, Systematic review, Innovation implementation, Healthcare organizations

## Abstract

**Background:**

Implementation and uptake of novel and cost-effective medicines can improve patient health outcomes and healthcare efficiency. However, the uptake of new medicines into practice faces a wide range of obstacles. Earlier reviews provided insights into determinants for new medicine uptake (such as medicine, prescriber, patient, organization, and external environment factors). However, the methodological approaches used had limitations (e.g., single author, narrative review, narrow search, no quality assessment of reviewed evidence). This systematic review aims to identify barriers and facilitators affecting the uptake of new medicines into clinical practice and identify areas for future research.

**Method:**

A systematic search of literature was undertaken within seven databases: Medline, EMBASE, Web of Science, CINAHL, Cochrane Library, SCOPUS, and PsychINFO. Included in the review were qualitative, quantitative, and mixed-methods studies focused on adult participants (18 years and older) requiring or taking new medicine(s) for any condition, in the context of healthcare organizations and which identified factors affecting the uptake of new medicines. The methodological quality was assessed using QATSDD tool. A narrative synthesis of reported factors was conducted using framework analysis and a conceptual framework was utilised to group them.

**Results:**

A total of 66 studies were included. Most studies (*n* = 62) were quantitative and used secondary data (*n* = 46) from various databases, e.g., insurance databases. The identified factors had a varied impact on the uptake of the different studied new medicines. Differently from earlier reviews, patient factors (patient education, engagement with treatment, therapy preferences), cost of new medicine, reimbursement and formulary conditions, and guidelines were suggested to influence the uptake. Also, the review highlighted that health economics, wider organizational factors, and underlying behaviours of adopters were not or under explored.

**Conclusion:**

This systematic review has identified a broad range of factors affecting the uptake of new medicines within healthcare organizations, which were grouped into patient, prescriber, medicine, organizational, and external environment factors. This systematic review also identifies additional factors affecting new medicine use not reported in earlier reviews, which included patient influence and education level, cost of new medicines, formulary and reimbursement restrictions, and guidelines.

**Registration:**

PROSPERO database (CRD42018108536).

**Supplementary Information:**

The online version contains supplementary material available at 10.1186/s12913-021-07196-4.

## Introduction

The uptake of an evidence-based intervention in clinical practice can take on average 17 years before it becomes part of a routine practice [1]. In healthcare, medicines are deemed to be the most common therapeutic intervention requiring significant funds from the system [2]. The slow uptake of cost-effective and novel medicines can delay improvements in patient health outcomes, healthcare efficiency, and even lessen the international competitiveness of the country in the life sciences sector [2–4]. For instance, in the United Kingdom (UK), the relative uptake of nationally recommended new medicines often lags behind other comparative countries’ health systems such as Australia, Canada or France [5].

There is a considerable amount of scientific literature exploring why the implementation of evidence-based interventions succeeds or fails within a complex healthcare environment [6]. Factors affecting implementation outcomes have been grouped into patient, provider, innovation, structural and organizational factors [7]. At the patient level, earlier reviews indicated patients’ socio-demographic and economic characteristics influenced the uptake of new medicines [8–10]. However, patients’ influence through their involvement in decision-making was relatively unexplored [8–10]. At provider level, prescribers’ scientific orientation and prescribing habits were suggested to affect uptake [10]. Furthermore, innovation level factors, such as effectiveness, safety-profile, convenience, and therapeutic novelty of new medicines were considered important aspects. Reviews concluded that cost was of low importance [8–10], but cost could be a factor in current healthcare systems as balancing increasing expenditure on medicines and available funding is becoming harder [2]. At an organizational level, mainly the impact of an organization’s characteristics, e.g., size, ownership, was suggested to have limited impact [8, 10]. Finally, structural level features, such as peer influence, pharmaceutical detailing, scientific literature and meetings, and regulatory pressures were identified as potential factors [8–10].

Although these earlier reviews provided some insight into the determinants of new medicine uptake, the methodological approaches had limitations (e.g., single author, narrative review, narrow search, no quality assessment of reviewed evidence). Also, healthcare systems have changed rapidly over the last ten years with increasing focus on patient-centred care and patient involvement in decision-making [11], use of medicines [2], expenditure on medicines [2], and new policies being developed to improve patient access to new medicines [12]. Studies in earlier reviews might not have captured all factors relevant to current healthcare systems and hence an updated review is warranted. This review, therefore, aims to identify barriers and facilitators affecting the uptake of new medicines into clinical practice, including areas for future research. Also, the review sought to provide more insight on the factors unexplored in earlier reviews such as patient influence and cost of new medicines.

## Methods

### Protocol and registration

A protocol for this review was registered on PROSPERO (Registration number: CRD42018108536) [13]. The conduct of the systematic review was guided by the Preferred Reporting Items for Systematic Review and Meta-Analysis (PRISMA) statement [14] (see Additional file 1 for the PRISMA checklist).

### Eligibility criteria

The inclusion criteria were established using the PICOS framework [15]. Eligible studies focused on adult participants (18 years and older) requiring or taking any new medicine(s) for any condition in the context of healthcare organizations. The World Health Organization definition of health innovation was used to define ‘new medicine’ as new or improved pharmaceutical product which improved people’s health and aimed to “add value in the form of improved efficiency, effectiveness, quality, sustainability, safety and/or affordability” [16]. Studied healthcare organizations were primary or secondary care. Eligible studies identified factors affecting (impeding or facilitating) the uptake of new medicines. The authors of this review considered uptake as the use of a new medicine within a healthcare organization within five years after it had been approved by the regulatory agency of the country where the study was conducted. Studies that only reported prescribing trends and/or patient demographics (age, gender) and clinical comorbidities were excluded. Qualitative, quantitative, or mixed-methods empirical studies published in English were eligible. Grey literature (conference proceedings, theses), review articles, clinical guidelines, and incomplete studies were excluded.

As healthcare systems have changed rapidly over the last ten years in relation to medicine use [2, 11, 12], studies from 2008 and onwards were included in the review to capture studies more relevant to current healthcare systems. Also, the review had a broad search strategy over seven databases, thus the time period limitation allowed to process a manageable number of studies yielded by the search.

### Information sources

The search was conducted in seven electronic databases: Medline, EMBASE, Web of Science, CINAHL, Cochrane Library, SCOPUS, and PsychINFO. Hand-searching was conducted using Google Scholar, reference lists, and forward citations of included studies and relevant systematic reviews to identify relevant studies that were inaccurately indexed or unindexed.

### Search

The search strategy was developed in collaboration with a subject librarian at the University of Bradford. The search was completed on 4 September 2018 and updated on 23 April 2020. The search terms were developed from four search categories: ‘uptake’, ‘new medicine’, ‘healthcare organization’, and ‘barriers and facilitators’ (see Additional file 2 for Medline search strategy).

### Study selection

After the removal of duplicates using the reference management software (EndNote X7®), one reviewer (KM) independently screened titles and abstracts. The second reviewer (JT) screened rejected articles after titles and abstract screening to minimise the removal of potentially relevant studies [17]. Two reviewers (KM, JT) independently reviewed full-texts of potentially relevant studies. The first reviewer (KM) screened the reference lists and forward citations, and the second reviewer (JT) independently reviewed studies deemed to meet the eligibility criteria. Any disagreements were discussed to reach a consensus. If consensus was not reached, the third reviewer (IM) reviewed disagreements.

### Data collection process and data items

A standardised proforma was developed by the research team and piloted with five studies before being finalised. One reviewer (KM) independently extracted data for 100% of the studies and the second reviewer (JT) independently checked the data extraction forms for accuracy and completeness. Any disagreements were discussed to reach a consensus. Abstracted data included citation information, study information (aim, design, data source, setting), studied new medicine, participant details, findings relevant to this review, funding source and reported conflict of interest. Lead authors of the studies were contacted via e-mail to provide missing or additional data if required.

### Risk of bias in individual studies

Two independent reviewers (KM and JT or IM) assessed risk of bias of included individual studies using the Quality Assessment Tool for Studies with Diverse Designs (QATSDD) [18]. The QATSDD tool consists of 16 criteria and is validated to assess studies with heterogeneous study designs. The following aspects of studies were examined: theoretical framework; aims and objectives; research setting; sample size and representativeness; data collection procedure and rationale; recruitment; appropriateness, reliability and validity of data analysis tools or process; user involvement; strengths and limitations. Reviewers scored each study on a scale of 0 (not at all/not stated) to 3 (complete/explicitly stated) against each criterion. The maximum score was of 42 for quantitative and qualitative studies and 46 for mixed-methods studies. Disagreements were resolved through discussion or by a third reviewer (KM, JT, or IM). The total score for each study was calculated by adding scores for each criterion and expressed as a percentage (0–100%). Studies with scores < 50% were classed as low, 50 to > 70% as moderate, and > 70% as high-quality studies. Although the low methodological quality studies were not excluded, they were given less weight in the synthesis of results and conclusions.

### Synthesis of results

The meta-analysis was not feasible due to the heterogeneity of study designs and methods used. Therefore, a narrative synthesis using a ‘best fit’ framework synthesis [19] was conducted to summarise the findings of reviewed studies.

‘Best fit’ framework synthesis is based on framework analysis [20] and is thought to bring a more transparent and pragmatic process than other more interpretive forms of synthesis [19]. It is a two-stage synthesis process. Firstly, an initial framework of preliminary themes was identified against which the data from the reviewed studies would be mapped. The initial framework used in this review was a multi-level framework by Chaudoir et al., which was developed by collecting implementation success factors for health innovations from multiple previous frameworks [7]. The themes in the Chaudoir et al. [7] framework were patient, provider, innovation, structural, and organizational.

The second stage of the ‘best fit’ framework synthesis involved reviewing the studies meeting the inclusion criteria and coding the identified factors affecting the uptake of new medicines against the themes in the multi-level framework by Chaudoir et al. [7]. The reviewing and summarising of the coded data were completed using NVivo11 software. New themes were created for data that could not be coded against the framework through a process of interpretation similar to thematic analysis [19].

Two reviewers (KM, IM) independently coded the material, and any discrepancies were resolved through discussion. Identified factors were finalised in a team discussion (KM, SR, KS, DP).

## Results

### Study selection

**Fig. 1:**
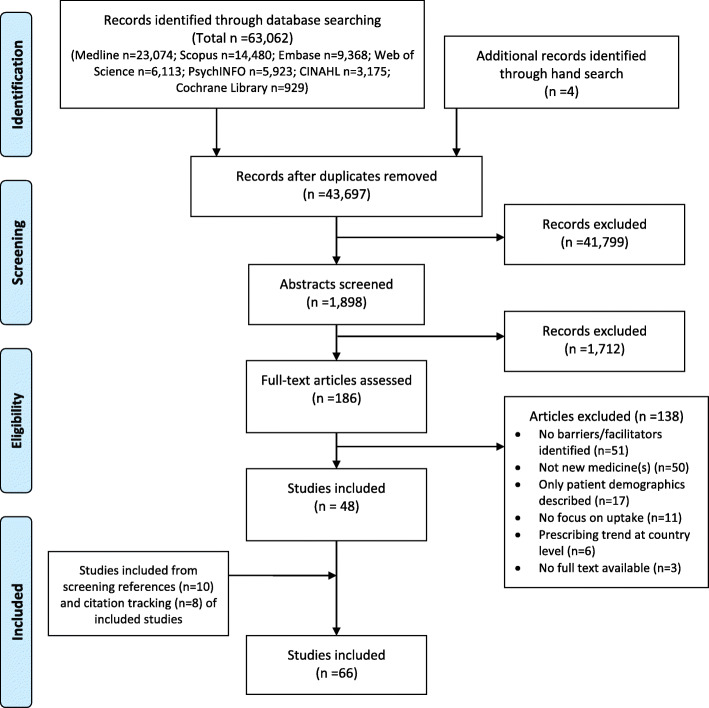
PRISMA flow diagram showing the systematic literature search and screening process

The study selection process is summarised in the PRISMA flowchart (Fig. 1). Of 43,697 unique citations identified in the search strategy, and an additional 22 studies retrieved through alternative methods, 66 studies were eligible for inclusion.

### Study characteristics

Study characteristic of included studies are presented in Table 1. Most of the studies (*n* = 62) used quantitative methods [21–49, 51–65, 68–79, 81–86]; three were qualitative [50, 67, 80], and one was mixed-methods [66]. The predominant source of data collection was secondary data (*n* = 46) from various databases and registries [22–25, 27, 29–34, 36, 40–44, 46, 48, 51–64, 66, 70–72, 74, 78, 79, 81–85, 87]. Other studies (*n* = 17) used surveys [26, 44, 47, 67, 68, 73], interviews [21, 26, 37, 50, 57, 66, 67, 76, 80], patients’ medical records [28, 45], prescriptions from community pharmacies [75], observations [35], or a focus group [80] to collect primary data. Two studies [38, 49] used both primary and secondary data. Studied new medicines were from twenty different therapeutic classes and five studies described medicines as newly marketed.

**Table 1:** Characteristics of included studies looking at factors affecting the uptake of new medicines

Author(s), publication year, country	Study objective related to this systematic review	Study Design	Data Source	Setting	Medicine(s)	Sample	Key Findings	Funding source and Conflict of interest (COI)
Abraham et al. (2010), USA [21]	To investigate if participation in clinical trials research network influences adoption of alcohol pharmacotherapies in publicly funded programs	Quantitative	Face-to-face interviews and brief telephone interviews	Primary and secondary care	acamprosate	244 public programs, 127 Clinical Trail Network (CTN) affiliated program administrators	Affiliation of programs with CTN; Percentage of master’s level counsellors; Access to a prescribing physician.	Funding: national funding body COI: not reported
AbuDagga et al. (2014), USA [22]	To identify factors associated with dabigatran versus warfarin use	Quantitative	Administrative pharmacy and medical claims database	Primary and secondary care	dabigatran	20,320 patients	Patient’s clinical and demographic characteristics; Speciality of prescriber; Patient’s health insurance plan type.	Funding: Daiichi Sankyo COI: one author was employee of Daiichi Sankyo; another received payments from Daiichi Sankyo; four authors-none
Anderson et al. (2015), USA [23]	To determine if conflict of interest policies influence psychiatrists’ antipsychotic prescribing and compare prescribing between academic and non-academic psychiatrists	Quantitative	IMS Health databases and physicians’ characteristics database	Primary and secondary care	Nine new and reformulated antipsychotics	2464 prescribers	Affiliation with academic medical centres with conflict-of-interest policies; Type of prescriber (academic or non-academic).	Funding: national funding body COI: none
Anderson et al. (2018), USA [24]	To explore characteristics of prescribers adopting new cardiovascular medicines	Quantitative	IMS Health databases	Primary and secondary care	dabigatran, aliskiren	5953 physicians	Speciality of prescriber; Gender of prescriber; Medical school attended by prescriber.	Funding: national funding body COI: none
Baik et al. (2016), USA [25]	To evaluate how patient characteristics are associated with the initiation of anticoagulant for patients newly diagnosed with atrial fibrillation	Quantitative	Pharmacy claims database	Primary and secondary care	dabigatran, rivaroxaban	17,193 patients	Patient clinical and demographic characteristics; Patient’s health insurance plan type; Out-of-pocket expenses- no effect.	Funding: national funding body COI: none
Boon et al. (2008), Belgium [26]	To examine the impact of reimbursement restrictions on the choice of antiepileptic (AEDs) medicines	Quantitative	Structured face-to-face interviews	Secondary care	16 AEDs, including old and new	100 neurologists	Reimbursement condition; Formulary restrictions.	Funding: GlaxoSmithKline COI: not reported
Bourke and Roper (2012), UK [27]	To explore the factors that shape the timing of the first prescription of six new medicines by General Practitioners (GPs)	Quantitative	Prescribing and GP characteristics databases	Primary care	escitalopram, rofecoxib, esomeprazole, desloratadine, nicotine, drospirenone and oestrogen	625 GP practices	Availability of nurse or clerical support; Participation in national incentive program to reduce prescribing costs; Previous early adoption of new medicines; GP’s prescribing portfolio size; Geographical location of GP practice.	Funding: not reported COI: not reported
Brais et al. (2017), Canada [28]	To identify predictors of oral anticoagulant choice for patients with atrial fibrillation	Quantitative	Electronic medical records	Secondary care	dabigatran, rivaroxaban, apixaban	439 patients at single teaching hospital	Patient’s demographic and clinical characteristics; Speciality of prescriber.	Funding: Bayer Inc., and Bristol-Myers Squibb Company-Pfizer alliance COI: not reported
Burden et al. (2015), Canada [29]	To examine the impact of formulary changes to the use of zoledronic acid	Quantitative	Pharmacy claim database and prescriber databases	Primary and secondary care	zolendronic acid, denosumab	18,226 patients	Formulary status change (removal of prior authorisation); Speciality of prescriber; Gender of prescriber.	Funding: national funding body COI: none
Carracedo-Martínez et al. (2017), Spain [30]	To assess the impact of the removal of prior authorization requirements for two coxibs on their use	Quantitative	Pharmacy claim database	Primary care	celecoxib, etoricoxib	One health district,catchment area of 383,125 people	Formulary prescribing conditions (prior authorisation requirement).	Funding: none COI: none
Chamberlain et al. (2014), UK [31]	To explore the impact of the Cancer Drug Fund (CDF) on access to cancer medicines in England, compared with Wales	Quantitative	IMS Health databases	Secondary care	15 cancer medicines	Not stated- prescribing volumes milligrams/1000 population used	The CDF was associated with higher prescription volumes in England for most medicines, which NICE had rejected for some or all indications pre-CDF and for medicines, which NICE had not appraised pre-CDF, but subsequently rejected.	Funding: national funding body COI: none
Chitagunta et al. (2009), USA [32]	To study the role of learning in the diffusion of three Cox-2 Inhibitors before withdrawal of rofecoxib	Quantitative	Prescription and advertising expenditure databases, published articles	Primary and secondary care	celecoxib, rofecoxib, valdecoxib	6577 patients and 17,329 prescriptions	Advertising, news and academic articles; Socio-economic status of patient; Patient’s demographic characteristics; Patient’s health insurance plan type; Patient’s satisfaction with existing treatment.	Funding: not reported COI: not reported
Chressanthis et al. (2012), USA [33]	To examine the effect of access limits to pharmaceutical representatives on new medicines prescribing by physicians	Quantitative	IMS Health databases	Primary and secondary care	sitagliptin	65,131 physicians	Organisation restrictions to pharmaceutical representative access; Speciality and age of prescriber; Size and geographical location of organisation	Funding: AstraZeneca COI: two authors were employees of AstraZeneca
Conti et al. (2012), USA [34]	To examines how evidence of the incremental effectiveness of novel chemotherapy medicines impacts on the adoption by physicians	Quantitative	Chemotherapy order system database	Secondary care	Seven oral chemotherapy medicines	4,344,711 patients, 122 medical oncology practices in 35 the USA states	Severity of the underlying disease; Clinical trials and media reports concurrent with market launch date; Medicine effectiveness.	Funding: national funding body COI: not reported
DeVore et al. (2018), USA [35]	To identified patient, provider, and practice characteristics associated with sacubitril/valsartan use	Quantitative	Observations	Primary and secondary care	sacubitril/valsartan	4216 patients, 121 sites across the USA	Patient’s clinical and demographic characteristics; Socio-economic status of patient; Patient’s health insurance plan type; Speciality of prescriber; Size of organisation; Staff composition at the organisation.	Funding: Novartis COI: five authors in previous receipt of funding from pharmaceutical industry; two acts as consultants to pharmaceutical industry; two were employees of Novartis
Donohue et al. (2018), USA [36]	To estimate the effect of peer adoption of three first-in-class medications on physicians’ own adoption of those medications.	Quantitative	IMS Health, insurance, and administrative claims databases	Primary and secondary care	dabigatran, sitigliptin, aliskiren	11,958 physicians	Peer influence (internal and external).	Funding: national funding body COI: not reported
Ducharme and Abraham (2008), USA [37]	To examine predictors of buprenorphine adoption	Quantitative	Brief telephone interviews and survey database	Primary and secondary care	buprenorphine	Staff members from 49 USA states and a data set of 12,236 substance abuse treatment facilities	Government owned and non-profit facilities; Hospital-based programs and opioid treatment programs; Programs offering detoxification services; Accredited programmes; Programmes serving adult population; Geographical location and size of programme; Government funding; Programs having at least one managed care contract; Coverage of medicine by patient’s health insurance.	Funding: national funding body COI: none
Dybdahl et al. (2011), Denmark [38]	To analyse associations between GPs’ clinical interests and their preference for new medicine	Quantitative	Postal survey and pharmacy prescription database	Primary care	Three COX-2 inhibitors and six angiotensin-II antagonists medicines	68 GPs	Continuous medical education activities.	Funding: not reported COI: authors received consultant fees or/and were previously involved in pharmaceutical industry funded research
Friedman et al. (2010), USA [39]	To examine the influence of senior managers’ characteristics on the adoption of buprenorphine	Quantitative	Telephone survey	Primary and secondary care	buprenorphine	547 pairs of administrative directors and clinical supervisors	Gender, age, the length of service and views of programme directors on treatment; Affiliations and accreditation of programme; Breadth of provided medical services and use of other medicines; Staff composition; Gender of patients.	Funding: national funding body COI:
Fuksa et al. (2015), Czech Republic [40]	To evaluate the overall changes in statin utilisation and expenditure with regards to the changing prescribing conditions	Quantitative	Insurance prescription claims database	Primary and secondary care	atorvastatin, rosuvastatin	774,281 patients	Changes in formulary prescribing conditions.	Funding: not reported COI: not reported
Garjon et al. (2012), Spain [41]	To analyse the diffusion of new medicines during the first months of use and examine the adoption between family physicians and specialists	Quantitative	Prescription database	Primary and secondary care	cefditoren, duloxetine, etoricoxib, ezetimibe, levocetirizine, olmesartan, pregabalin and tiotropium	1248 physicians	Speciality of prescriber; Therapeutic innovation of medicine; Range of indications for medicine; Prior authorisation requirement.	Funding: not reported COI: three authors received educational fees from pharmaceutical industry
Groves et al. (2010), Canada [42]	To assess relationship between physicians’ characteristics and prescribing of new medicines	Quantitative	Administrative and insurance claims databases	Primary and secondary care	Four COX-2 inhibitors and two non-selective NSAIDs medicines	925 physicians	Demographic characteristics; Speciality of prescriber; Geographical location of practice; Caseload of prescriber.	Funding: not reported COI: not declared
Haider et al. (2008), Sweden [43]	To examine the association between educational level of patients and the use of newly marketed medicines among elderly patients	Quantitative	Three national registers: prescribed medicines, inpatient, and education	Primary and secondary care	18 newly marketed medicines with at least 350 users	626,258 patients	Patient’s educational level and gender; Number of prescribed medicines for patient; Patient’s residential area.	Funding: not reported COI: none
Hickson et al. (2019), USA [44]	To describe trends over time in the initiation of the dipeptidyl peptidase-4 (DPP-4) inhibitors before and after removal of the rosiglitazone black box warning and restricted access program	Quantitative	Administrative claims database	Primary and secondary care	DPP-4 inhibitors	280,969 patients	Regulatory restrictions to the use of medicines in the same category as new medicines.	Funding: not reported COI: one author was employee of Truven Health Analytics/IBM Watson Health
Hirunrassamee and Ratanawijitrasin (2009), Thailand [45]	To assess access to medicines and other medical technologies under the three government health insurance schemes	Quantitative	Hospital electronic database and paper records	Secondary care	Antiepileptic and antineoplastic lung cancer medicines	913 patients (antiepileptics), 33 patients (antineoplastics); 3 hospital sites	Patient’s health insurance plan type; Out-of-pocket payments.	Funding: not reported COI: not reported
Hsieh and Liu (2012), Taiwan [46]	To explore issues surrounding utilisation of biologics in Taiwan	Quantitative	National insurance claims database	Secondary care	trastuzumab, rituximab, peginterferon-alfa-2A, etanercept	590 patients	Size of hospital; Type of hospital ownership; Patient’s clinical characteristics.	Funding: national funding body, Johnson & Johnson COI: none
Huang et al. (2013), USA [47]	To examine factors that influence doctors’ decision in initiating or switching from warfarin to dabigatran	Quantitative	Online survey	Secondary care	dabigatran	65 physicians	Cost of medicine; Patient’s socioeconomic status; Patient’s clinical characteristics; Speciality of prescriber; Experience of prescriber with the medicine; Perceived benefits of new over ‘old’ therapy.	Funding: not reported COI: not reported
Huskamp et al. (2013), USA [48]	To examined physician adoption of second-generation antipsychotic medications and identified physician-level factors associated with early adoption	Quantitative	IMS Health prescription database	Primary and secondary care	olanzapine, quetiapine, ziprasidone, and aripiprazole	30,369 physicians	Age and gender of prescriber; Speciality of prescriber; Size and type of practice; Caseload of prescriber; Medical school location of prescriber.	Funding: national funding body COI: one author consulted National Railways Labor Conference
Iyengar et al. (2011), USA [49]	To assess the impact of social networks on the adoption of a new medicine by physicians	Quantitative	Mailed and online survey, IMS Health databases, and pharmaceutical company sales calls records	Primary and secondary care	A newly launched prescription medicine used to treat a specific type of viral infection (short and long-term)	185 physicians from three cities	Peers influence- the level of impact is shaped by peer’s usage volume and by the clinicians’ perception of their self-reported opinion leadership. Perceived leaders by colleagues adopted new medicine quicker than self-reported leaders.	Funding: not reported COI: not reported
Karampli et al. (2020), Greece [50]	To explore factors influencing adoption of new antidiabetic medicines for patients with type 2 diabetes mellitus	Qualitative	Semi-structured face-to-face interviews	Primary and secondary care	DDP-4 inhibitors, GLP-1 agonists, SGLT2 inhibitors, new oral fixed-dose combinations of glycose-lowering medications, new dosage forms	10 physicians	New medicine’s safety profile, efficacy, degree of relative advantage, formulation, cost, ease of use; Habitual prescribing of physician; Physician’s needs and values of practice; Physician’s experience with established medicines; Patient’s clinical and demographic characteristics; Patient’s preferences and adherence to treatment; Patient’s health insurance plan type; Working place of physician.	Funding: none COI: none
Keating et al. (2018), USA [51]	To examine diffusion of bevacizumab and assess variation in use across oncology practices	Quantitative	Insurance claim database	Secondary care	bevacizumab	2329 practices	Size and accreditation of organisation; Staff composition at organisation; Patient’s clinical and demographic characteristics; Patient’s socio-economic status.	Funding: national funding body COI: none
Keating et al. (2020), USA [52]	To understand adoption of bevacizumab by oncologists for patients with cancer using network analysis method	Quantitative	Insurance claim database	Secondary care	bevacizumab	44,012 patients, 3261physicians, 51 hospital referral regions	Patient’s clinical and demographic characteristics; Age of prescriber; Peer influence.	Funding: national funding body COI: one author received consultant fees from Grail
Kennedy et al. (2020), Ireland [53]	To compare the use of direct oral anticoagulants in areas with warfarin clinics compared to those without	Quantitative	Pharmacy claims database shapefiles of warfarin clinics and areas	Primary care	apixaban, dabigatran, edoxaban, rivaroxaban		Presence or absence of hospital-based warfarin clinics- no effect.	Funding: national funding body COI: none
Kereszturi et al. (2015), Hungary [54]	To identify socio-demographic, workplace, practice, prescribing and patient characteristics of the early prescribers of the newly marketed innovative medicines	Quantitative	DoktorInfo prescription database	Secondary care	vildagliptin with metformin and metformin with sitagliptin combinations	318 physicians	Portfolio width and prescribing volume of prescriber; Number of patients looked after by prescriber and number of consultations per patient; Prescribing of other branded medicines; Proportion of patients treated with insulin.	Funding: AXA Research Fund COI: not reported
King et al. (2013), USA [55]	To examine the effect of attending a medical school with an active policy on restricting gifts from representatives of pharmaceutical and device industries on subsequent prescribing behaviour	Quantitative	IMS Health database and physicians’ characteristics database	Primary care	lisdexamfetamine, paliperidone, desvenlafaxine	8602 physicians	Attending a medical school with an active gift restriction policy; Length of exposure to gift restriction policy.	Funding: national funding body COI: none
King and Bearman (2017), USA [56]	To examine how different pharmaceutical detailing regulations and peer influence shaped medicine diffusion processes of newly marketed medicines	Quantitative	IMS Health prescription database	Primary care	lisdexamfetamine, duloxetine	208,072 physicians for duloxetine, 215,445 physicians for lis-dexamfetamine	Policies limiting or banning gifts from pharmaceutical industry; Peer influence.	Funding: not reported COI: not reported
Knudsen et al. (2009), USA [57]	To examines the adoption of buprenorphine over a 2-year period in community-based treatment programs associated and not with Clinical Trials Network (CTN)	Quantitative	Telephone and face-to-face interviews	Primary care	buprenorphine	193 community-based treatment programs (CTPs)	Involvement in CTN buprenorphine protocol development; Size of organisation; Access to prescribers; Offering other inpatient services; Type of organisation.	Funding: national funding body COI: not reported
Lin H et al. (2011), USA [58]	To explore the patterns of physician prescribing and medication choice for major depressive disorder between 1993 and 2007	Quantitative	National survey database	Primary care	Four antidepressant drug classes	125,605,444 patients	Patient’s health insurance type; Age of patient; Practice geographical location	Funding: not reported COI: not reported
Lin S et al. (2011), Taiwan [59]	To examine how the prescribing decisions made by psychiatrists’ colleagues influence the likelihood of the psychiatrists’ initial prescription	Quantitative	National insurance database	Secondary care	duloxetine	155 psychiatrists	Speciality of prescriber; Clinical experience of prescriber; Adoption behaviour of colleagues.	Funding: university funding COI: not reported
Liu et al. (2011), Taiwan [60]	To investigate the effect of various economic factors on the diffusion of new medicines	Quantitative	National drug claims database	Primary and secondary care	seven oral anti-glycaemic medicines	3,384,223 prescriptions	Degree of competition in the pharmaceutical and health service market; Size of the provide; Type of organisation; Disease severity; Geographical location of organisation.	Funding: national funding body COI: not reported
Liu and Gupta (2012), USA [61]	To analyze individual physicians’ adoption of a newly launched prescription medicine	Quantitative	ImpactRx market research database and TNS Media Intelligence data (journal advertising expenditure)	Primary and secondary care	A newly launched medicine from one of the largest therapeutic classes of prescription medicines in USA, novel mechanism of action	2129 physicians	Targeted detailing, journal advertising, meetings and events sponsored by industry, peer influence, and patient requests has positive impact. Specialists and prescribers with larger prescription volumes in the studied therapeutic class and who practice in communities with a larger percentage of patients from a White background adopted the new medicine quicker.	Funding: not reported COI: not reported
Lo-Ciganic et al. (2016), USA [62]	To examine the physician adoption of dabigratran	Quantitative	IMS Health database and physicians’ characteristics database	Primary and secondary care	dabigatran	3911 prescribers	Speciality of prescriber; Prescribers age; Hospital referral region; Patient’s health insurance plan type.	Funding: national funding body; university funding COI: none
Luo et al. (2017), USA [63]	To assess the prevalence and variation in sacubitril/valsartan prescription among a real-world population with heart failure with reduced ejection fraction	Quantitative	National registry of hospitalised patients	Secondary care	sacubitril/valsartan	21,078 patients, 241 hospital sites	Geographical location of organisation; Accreditation of organisation-no effect; Patient’s clinical and demographic characteristics; Patient’s health insurance plan type-no effect.	Funding: Novartis COI: one author was employee and three received consultant fees from Novartis; one received research funding from pharmaceutical companies
Luo et al. (2018), USA [64]	To evaluate the early impact of this national treatment guideline update on the use of sacubitril/valsartan	Quantitative	National registry of hospitalised patients and national hospitals survey database	Secondary care	sacubitril/valsartan	7200 patients	Size, location, accreditation of organisation and available services- no effect; National guideline publication- little/no effect.	Funding: Novartis COI: one author was employee of Novartis; four received research support from industry
Luo et al. (2019), USA [65]	To identify hospital characteristics associated with the use of sacubitril/valsartan	Quantitative	National registry of hospitalised patients; national hospitals survey database, US census region, insurance claim database.	Secondary care	sacubitril/valsartan	16,674 patients, 210 hospital sites	Size and accreditation of organisation-no effect; Organisation type (profit/non-profit); Geographical location of organisation; Follow-up ambulatory services-no effect.	Funding: Novartis COI: one author was employee of Novartis; three authors received research support from pharmaceutical companies
Manchanda et al. (2008), USA [66]	To explore impact of marketing and interpersonal communication on the adoption of a new medicine in two unrelated markets	Mixed-methods	Pharmacy audit database, pharmaceutical company marketing records, interviews	Primary and secondary care	A new medicine from important medicine category	466 physicians	Pharmaceutical industry targeted communication; Detailing, detailing stock, and sampling stock by pharmaceutical industry; Peer influence; Direct advertising to patients-no effect.	Funding: university funding COI: not reported
Martin et al. (2017), France [67]	To explore the barriers to the diffusion of newly released oral targeted therapies dedicated to metastatic breast cancer	Qualitative	Semi-structured face-to-face interviews	Secondary care	everolimus	40 physicians	Amount of new information to be acquired about the medicine; Lack of organisation in patient management; Time required to manage oral cancer treatments; Prescriber’s prescribing habits; No clear position of the new medicine in the therapeutic strategy; Being the only oncologist or multi-organ oncologist in the organisation.	Funding: Odyssea association COI: none
Murphy et al. (2018), Ireland [68]	To explore factors that influence general practitioners prescribing of direct oral anticoagulants	Quantitative	Postal survey	Primary care	apixaban, dabigatran, edoxaban, rivaroxaban	221 general practitioners	Hospital colleagues’ influence; Local and national guidelines; Conferences and journal articles; Clinical and demographic characteristics of patient; Perceived efficacy of medicine; Monitoring requirements; Size of practice.	Funding: none COI: none
Netherland et al. (2009), USA [69]	To examine factors affecting willingness to adopt buprenorphine by physicians	Quantitative	On-site and online surveys	Primary care	buprenorphine	172 prescribers, two national programs	Training of clinical staff on new medicine; Access to other services and treatments; Presence of effective referral system for alternative treatment; Adequate time per visit; Patients’ concerns about medicine; Availability of clinical guidelines and medicine; Reimbursement for consultation; Record keeping requirements; Access to an expert prescriber; Gender and ethnicity of prescriber; Experience and speciality of prescriber.	Funding: national funding body COI: not reported
Ohl et al. (2013), USA [70]	To determine rural-urban variation in adoption of raltegravir amongst in national Veterans Affairs healthcare	Quantitative	Health care and residence databases	Primary and secondary care	raltegravir	1222 patients	Residential area of patient; Patient’s clinical and demographic characteristics; Previous use of antiretroviral medicines.	Funding: national funding body COI: not reported
Ohlsson et al. (2009), Sweden [71]	To investigate determinants of early adoption of rosuvastatin	Quantitative	National drug register	Primary care	rosuvastatin	73,547 prescriptions from 170 health care practices	Type of ownership; Existence of strong therapeutic traditions; Socioeconomic status of patient.	Funding: not reported COI: not reported
Patel et al. (2015), USA [72]	To characterise the prevalence, patterns, and predictors of direct oral anticoagulants versus warfarin therapy at discharge among atrial fibrillation patients hospitalised with ischemic stroke or transient ischemic attack	Quantitative	National stroke database	Secondary care	dabigatran, rivaroxaban	61,655 patients from 1542 hospitals	Patient’s clinical characteristics; Ambulatory status of patient; Discharge destination; Patient’s health insurance plan type.	Funding: national funding body COI: two authors received consultant fees and three research support from pharmaceutical industries
Potpara et al. (2017), Balkan countries [73]	To explore the use of direct oral anticoagulants in seven Balkan countries	Quantitative	Online survey	Secondary care	dabigatran, rivaroxaban, apixaban	2663 patients from 49 centres	Speciality of prescriber; Patient’s clinical characteristics; Atrial fibrillation treatment strategy; Hospital-based centres; Previous use of oral anticoagulants.	Funding: none COI: six authors received speaker fees and one consultant fees from pharmaceutical industry
Rodwin et al. (2020), USA [74]	To examine patient and hospital-level factors associated with prasugrel and ticagrelor use in acute myocardial infarction	Quantitative	National hospital registry for patients with myocardial infarction	Secondary care	prasugrel, ticagrelor	362,354 patients, 801 hospitals	Patient’s clinical and demographic characteristics; Patient’s health insurance plan type; Number of patients treated in hospital; Geographical location and accreditation of organisation; Speed of adoption of previous innovation.	Funding: national funding body COI: one author received consultant fees and research support for pharmaceutical industry; one author received salary support from the funding body, funding from insurance companies, and hold equity interest in Medtronic
Sato et al. (2012), Japan [75]	To assess the impact of the sitagliptin regulatory safety alert on the prescribing behaviour	Quantitative	Prescription data from 300 pharmacies	Primary and secondary care	sitagliptin	87,678 patients	Size of hospital; Speciality of prescriber; Safety alert.	Funding: none COI: two authors received research support from pharmaceutical industry
Savage et al. (2012), USA [76]	To examine the extent to which programs’ interorganisational institutional and resource-based linkages predict the likelihood of being an earlier adopter, later adopter, or non-adopter of buprenorphine	Quantitative	Face-to-face interviews and brief telephone interviews	Primary and secondary care	buprenorphine	345 privately funded substance abuse treatment programs	Membership in national and regional associations; Detailing activities by pharmaceutical companies; Use of National Institute on Drug Abuse website as an information source.	Funding: national funding body COI: not reported
Scholten et al. (2015), Germany [77]	To examine the factors at the organisational level that influence the implementation of systemic thrombolysis in stroke patients.	Quantitative	Hospital structure quality reports registry	Secondary care	alteplase	286 hospitals	Existence of stroke unit; Hospital size.	Funding: none COI: none
Steinberg et al. (2013), USA [78]	To identify patient and/or provider factors associated with the use of dabigatran in patients with atrial fibrillation	Quantitative	National registry for outpatients with atrial fibrillation	Secondary care	dabigatran	8794 patients, 176 sites	Patient’s clinical and demographic characteristics; Patient’s health insurance plan type; Education level of patient; Current antiarrhythmic use; Speciality of prescriber.	Funding: Janssen Scientific Affairs, national funding body COI: seven author received consultant fees, five research support, two speaker fees from pharmaceutical industry, one author employed by Johnson & Johnson.
Tanislav et al. (2018), Germany [79]	To investigate oral anticoagulation in stroke patients documented in a nationwide registry	Quantitative	National hospital quality registry	Secondary care	apixaban, dabigatran, edoxaban, rivaroxaban	3813 patients	Treatment on stroke unit; Patient’s clinical and demographic characteristics; Previous oral anticoagulant/ antiplatelet use.	Funding: none COI: none
Tobin et al. (2008), Australia [80]	To identify the factors that influence prescribing of new medicines among general practitioners, endocrinologists and psychiatrists	Qualitative	Focus groups with semi-structure interview guide	Primary and secondary care	Medicine that has in the past 1–2 years been in Pharmaceutical Benefit Scheme (PBS) listed, or released to the market, or a new chemical entity	21 prescribers	Socioeconomic status of patient; Clinical need for medicine; New medicine’s attributes: adverse effects, safety, efficacy; Listing of medicine in PBS; Peer influence; Prescriber’s familiarity with the therapeutic area; Prescriber’s knowledge of the medicine.	Funding: non-profit organisation COI: not reported
Tsai et al. (2010), Taiwan [81]	To examine factors affecting thiazolidinediones penetration into Taiwan’s hospitals	Quantitative	National health insurance database	Secondary care	pioglitazone, rosiglitazone	580 hospitals	Degree of competition in the pharmaceutical market; Type of hospital; Type of ownership of hospital; Geographical location of hospital; Cost of medicines; Prescribing volume of diabetic medicines by hospital.	Funding: national funding body COI: not reported
Wang et al. (2010), Taiwan [82]	To determine if socioeconomic status impacts adoption of newly reimbursed non-steroidal anti-inflammatory medicines under a universal health insurance program	Quantitative	Eight different electronic databases	Primary and secondary care	rofecoxib, celexocib, nimesultide	875 patients	Patient’s clinical and demographic characteristics; Patient’s socio-economic status; Patient’s habits of health-care utilisation.	Funding: not reported COI: not reported
Weir et al. (2012), Canada [83]	To explore the impacts of formulary listing changes and regulatory agency warnings on the use of erythropoiesis-stimulating agents in cancer patients	Quantitative	Prescription and physician characteristics databases, province people registry	Secondary care	Three erythropoiesis-stimulating agents	171,967 patients	Formulary changes in reducing or removing restrictions for use; Safety warnings from regulatory agencies.	Funding: national funding body COI: one author received honorarium from Amgen
Wen et al. (2011), Taiwan [84]	To characterise how a new medicine class for diabetes mellitus diffused in the health care market	Quantitative	National insurance claim database	Secondary care	rosiglitazone, pioglitazone	580 hospitals	Accreditation and type of hospital; Type of ownership of hospital; Degree of competition in the pharmaceutical market; Geographical location of hospital; Number of prescribers prescribing these medicines; Prior anti-diabetic prescription capacity.	Funding: national funding body COI: none
Zhang et al. (2019), Australia [85]	To evaluate how physicians’ risk preferences and personality affects their decisions to adopt new prescription medicines	Quantitative	Database of national panel survey of medical practitioners, insurance claim database	Primary care	apixaban, dabigatran, rivaroxaban	576 GPs	Socio-demographic characteristics of prescriber; Prescribing volume; Willingness to take clinical risks; Employment status in the GP practice; Time spent in consultations; Location of GP practice; GP practice affiliations and social practice characteristics-no effect; Patient’s demographic characteristics; Patient’s socio-economic status.	Funding: national funding body, university COI: none
Zhang et al. (2020), China [86]	To obtain information on the use of PD-1/PD-L1 inhibitors by oncologists in China	Quantitative	Online and offline survey	Secondary care	PD-1/PD-L1 checkpoint inhibitors	588 oncologists	Knowledge and understanding mechanism of action of new medicines; Experience in using new medicines; New medicine’s attributes: cost, efficacy, adverse effects.	Funding: none COI: none

### Risk of bias within studies

The methodological quality of studies ranged from 45 to 81%, with a mean score of 67% (see Additional file 3, Table S1). Two studies were deemed to be low, 38 medium, and 26 high quality. The most prominent methodological weaknesses were lack of reporting reliability and validity of data measurement tools used in quantitative studies and reliability of analytical process used in qualitative studies. There was no evidence of pilot testing or user involvement across all studies (Table 2).

**Table 2:** Summary of the scores for the 16 criteria used to assess the methodological quality shown for all studies, quantitative and qualitative studies*

QASTDD tool criteria and study design	Range	Mean	Standard deviation	% maximum of possible score achieved
1. **Explicit theoretical framework (all studies)**	**0–3**	**1.9**	**0.9**	**62%**
*Quantitative studies*	*0–3*	*1.9*	*0.9*	*62%*
*Qualitative studies*	*1–3*	*1.7*	*1.2*	*56%*
2. **Statement of aims/objectives in main body of report (all studies)**	**2–3**	**2.8**	**0.4**	**93%**
*Quantitative studies*	*2–3*	*2.8*	*0.4*	*92%*
*Qualitative studies*	*3*	*3*	*0*	*100%*
3. **Clear description of research setting (all studies)**	**2–3**	**2.7**	**0.5**	**89%**
*Quantitative studies*	*2–3*	*2.7*	*0.5*	*89%*
*Qualitative studies*	*2–3*	*2.7*	*0.5*	*89%*
4. **Evidence of sample size considered in terms of analysis (all studies)**	**0–3**	**1.5**	**0.7**	**50%**
*Quantitative studies*	*1–3*	*1.5*	*0.6*	*49%*
*Qualitative studies*	*0–3*	*2*	*1.7*	*67%*
5. **Representative sample of target group of a reasonable size (all studies)**	**1–3**	**2.2**	**0.5**	**74%**
*Quantitative studies*	*1–3*	*2.2*	*0.5*	*75%*
*Qualitative studies*	*2*	*2*	*0*	*67%*
6. **Description of procedure for data collection (all studies)**	**1–3**	**2.4**	**0.7**	**81%**
*Quantitative studies*	*1–3*	*2.4*	*0.7*	*81%*
*Qualitative studies*	*2–3*	*2.7*	*0.6*	*89%*
7. **Rationale for choice of data collection tool(s) (all studies)**	**0–3**	**1.7**	**0.9**	**58%**
*Quantitative studies*	*0–3*	*1.8*	*0.8*	*59%*
*Qualitative studies*	*0–2*	*0.7*	*1.2*	*22%*
8. **Detailed recruitment data (all studies)**	**0–3**	**2.0**	**1.0**	**67%**
*Quantitative studies*	*0–3*	*2.0*	*1.0*	*68%*
*Qualitative studies*	*0–3*	*1.7*	*1.5*	*56%*
9. **Statistical Assessment of reliability and validity of measurement tool(s) (Quantitative studies only)**	**0–3**	**0.9**	**1.1**	**29%**
10. **Fit between stated research question and method of data collection (Quantitative studies only)**	**2–3**	**2.8**	**0.4**	**93%**
11. **Fit between stated research question and format and content of data collection tool e.g. interview schedule (Qualitative studies only)**	**2–3**	**2.3**	**0.5**	**75%**
12. **Fit between research question and method analysis (all studies)**	**2–3**	**2.9**	**0.3**	**97%**
*Quantitative studies*	*2–3*	*2.9*	*0.2*	*98%*
*Qualitative studies*	*2–3*	*2.3*	*0.6*	*78%*
13. **Good justification for analytical method selected (all studies)**	**0–3**	**2.4**	**0.8**	**81%**
*Quantitative studies*	*0–3*	*2.5*	*0.8*	*83%*
*Qualitative studies*	*2–3*	*2.7*	*0.6*	*89%*
14. **Assessment of reliability of analytical process (Qualitative studies only)**	**0–2**	**1.0**	**0.8**	**33%**
15. **Evidence of user involvement in design (all studies)**	**0**	**0**	**0**	**0%**
16. **Strengths and limitations critically discussed (all studies)**	**0–3**	**1.9**	**0.7**	**63%**
*Quantitative studies*	*0–3*	*1.9*	*0.7*	*63%*
*Qualitative studies*	*1–2*	*1.3*	*0.6*	*44%*

*Quantitative studies n = 62, qualitative studies n = 3, mixed-methods study *n* = 1. As there was only one mixed-methods study, its scores were included in reporting score for all studies and individual scores can be found in Additional file 3, Table S1

### Synthesis of results

Factors affecting the uptake of new medicines were grouped into five thematic areas: patient, prescriber, medicine, organizational and external environment factors. Summary of main thematic areas with subthemes are shown in Table 3.

**Table 3:** Summary of main thematic areas and developed subthemes of factors affecting the uptake of new medicines

Thematic area	Subthemes
Patient-level factors	Demographic characteristics
	Socioeconomic status
	Health status
	Patient engagement with treatment
Prescriber factors	Socio-demographic characteristics
	Scope of expertise
	Knowledge and prescribing habits
Medicine-level factors	Efficacy
	Safety profile
	Cost
	Therapeutic innovation
	Medicine administrative burden
Organizational-level factors	Ownership status
	Teaching status
	Size
	Location
	Available services and resources
	Staff composition
	Care co-ordination and quality
External environment-level factors	Pharmaceutical detailing
	Reimbursement conditions and formulary status
	Peer influence (internal and external)
	Guidelines
	Other information sources
	Organization affiliations

The thematic area(s) in each included study is reported in Additional file 4, Table S2. External environment, organizational, patient and prescriber factors were reported most frequently (*n* = 36, *n* = 34, *n* = 31 and *n* = 29 studies respectively) and medicine factors (*n* = 18) were the least. Summary of factors affecting the uptake of new medicines referred to in the reviewed studies is displayed in Table 4.

**Table 4:** Summary of factors affecting the uptake of new medicines referred to in the reviewed studies

Identified factor	Number of studies referred to the factor	As facilitator	As barrier	No impact	Citations
**Patient factors**					
Age (younger)	18	11	4	3	[22, 25, 28, 32, 35, 51, 52, 58, 60, 63, 70–74, 78, 79, 82, 85]
Gender (male)	12	4	1	7	[22, 28, 29, 58, 60, 71–74, 78, 79, 82]
Ethnicity (White)	10	6	1	3	[25, 35, 51, 58, 61, 70, 72, 74, 78, 82]
Education level (higher)	5	4		1	[32, 35, 43, 78, 82]
Income (higher)	11	11		1	[22, 25, 32, 45, 47, 50, 51, 71, 80, 82, 85]
Insurance (private or more comprehensive)	9	9			[22, 25, 35, 45, 58, 62, 72, 74, 78]
Residential area (urban or more affluent)	3	3			[43, 70, 85]
Health condition (more severe & comorbidities)	13	5	8		[22, 28, 34, 35, 46, 51, 63, 72–74, 78, 79, 82]
Polypharmacy	9	3	4		[22, 25, 28, 43, 72, 73, 79]
Patient satisfaction, adherence to current therapy & monitoring	4	4			[32, 47, 50, 80]
Response to current therapy (poor)	3	3			[47, 70, 80]
Patients request & therapy preferences	5	5			[47, 50, 61, 69, 80]
**Prescriber factors**					
Age (younger)	7	4	2	3	[27, 29, 33, 42, 48, 53, 62]
Gender (male)	6	4		2	[23, 29, 42, 48, 62, 85]
Graduating from a top-20 medical or foreign school	3	3		2	[23, 48, 62]
Principal or partner GP	1	1			[85]
Specialist or secondary care prescriber	16	13	4	1	[22, 24, 28, 29, 33, 35, 41, 42, 47, 48, 59, 61, 62, 73, 75, 78]
Non-academic prescriber	1	1			[23]
Greater prescribing volume or portfolio breadth	5	5			[27, 48, 54, 61, 85]
Knowledge of new medicine	7	7			[47, 50, 67–69, 80, 86]
Continuing medical education activities	1	1			[38]
Early adopter in the past	1	1			[27]
Taking clinical risks & spending less time in consultations	1	1			[85]
**Medicine factors**					
Efficacy	6	6			[34, 50, 68, 69, 80, 86]
Safety concerns (adverse & long-term effects)	6		6		[44, 47, 50, 68, 80, 86]
Interactions with food/medicines (less)	3	3			[47, 68, 80]
High unit cost	5	5		3	[47, 50, 68, 80, 86]
Therapeutic innovation	5	5			[41, 48, 50, 60, 80]
Ease of use & administration	4	3	1	1	[47, 50, 69, 80]
Reduced monitoring & clinic visits	2	2			[47, 68]
**Organizational factors**					
Ownership status (private)	10	7	2	1	[21, 37, 46, 57, 60, 64, 65, 71, 81, 84]
Teaching status	8		1	7	[54, 63–65, 72–74, 77]
Size (larger)	17	11	3	3	[33, 35, 37, 46, 48, 51, 57, 60, 65, 68, 72, 75–77, 81, 82, 84]
Location (more populated)	10	3	3	5	[27, 42, 57, 63, 71–73, 77, 84, 85]
Availability of supportive services	11	7		4	[27, 35, 37, 39, 53, 57, 65, 69, 76, 77, 79]
Limited consultation time	2		2		[67, 69]
Number of specialists, nurses, or healthcare professionals (higher)	8	8			[21, 35, 39, 48, 51, 57, 59, 67]
Care co-ordination (fragmented)	2		2		[67, 69]
**External environment factors**					
Pharmaceutical detailing	11	11		1	[23, 33, 50, 55, 61, 66, 68, 76, 80]
Formulary or reimbursement restrictions	10		10		[23, 26, 29–31, 37, 40, 50, 80, 83]
Peer influence (internal & external)	14	14			[36, 49, 50, 52, 56, 59, 61, 64, 66, 68, 71, 80, 81, 84]
Recommended by guideline (international, national, or local)	6	5		1	[50, 64, 67–69, 86]
Scientific literature, websites, & conferences	6	6	1		[32, 34, 50, 68, 76, 80]
Organizational affiliations	6	4	2		[21, 39, 50, 51, 57, 76]

### Patient-level factors

#### Demographic characteristics (*n* = 21)

Studies reported mixed results of patients’ age, gender, and ethnicity impact on the uptake of new medicines. Some prescribers tended to prescribe new medicines to younger [22, 28, 35, 51, 52, 58, 63, 71, 72, 74, 78], male [22, 72, 74, 79], female [82], or white ethnicity [61] patients. Others observed use of new medicine in older patients [25, 32, 70, 82, 85], mixed impact of ethnicity [25, 35, 51, 72, 74, 78], or suggested that patients’ age [60, 73, 79], gender [28, 29, 58, 60, 71, 73, 78], ethnicity [58, 70, 82] had no impact on prescribing decisions. Studies were medium to high quality and one study [79] was low quality.

#### Socioeconomic status (n = 21)

Some prescribers reported that patients’ socioeconomic factors [68], which included education, income, health insurance plan, residential area, influenced their prescribing decisions. Some findings suggested patients with a higher level of education were more likely to receive new medicines [32, 35, 43, 78], regardless of their age, gender, education, type of residential area, number of medicines used, and comorbidity [43]. However, one study [82] observed no impact of patient education level. All studies were high-quality. Prescribers also considered affordability of new medicines by patients [50]. Some studies suggested patients with higher income or ability to pay out-of-pocket expenses were more likely to receive a new medicine [22, 32, 45, 47, 51, 71, 80, 82, 85], but one study observed no difference [25]. Only three studies were high-quality [32, 71, 82]. Furthermore, patients with private health insurance plans covering prescription medicines and medical care services were reported to have a greater access to new medicines [22, 25, 35, 45, 58, 62, 72, 74, 78]; two studies were high-quality [35, 78]. Lastly, some studies indicated that patients living in a capital city [43], urban [70], or more affluent areas [85] were more likely to receive new medicines; two studies were high-quality [43, 70].

#### Health status (*n* = 21)

Prescribers highlighted that patients’ clinical characteristics and comorbidities influenced new medicines use [50]. Some prescribers reported prescribing new medicines for patients with more severe disease [34, 35, 46, 63, 82] or polypharmacy [22, 25, 43]; five studies were high-quality [34, 35, 43, 46, 82]. Other low to high quality studies reported new medicines use in patients with fewer comorbidities, or less severe conditions [22, 28, 51, 72–74, 78, 79], and no polypharmacy or concomitant use of medicines increasing the risk of side effects [28, 72, 73, 79]. Medium to high quality studies reported patient’s poor response to the current treatment encouraged [47, 70, 80] and that patient’s satisfaction with the existing treatment discouraged new medicine use [32].

#### Patient engagement with treatment (*n* = 5)

Some prescribers stated that patients’ requests for a new medicine [47, 61, 80] and interest in it [67], adherence to current treatment [50, 80] and monitoring [47] were influential in prescribing decisions. Some prescribers were described as aiming for a shared decision-making thus patients’ therapy preference and compatibility with their lifestyle [50] shaped prescribing decisions. Only one study was high-quality [61].

### Prescriber factors

#### Socio-demographic characteristics (*n* = 11)

Medium to high quality studies suggested younger [33, 48, 53, 62] or older [27, 42], male [23, 42, 48, 85], graduating from a top-20 medical [23, 62] or foreign medical school [48] prescribers were earlier adopters. Other medium to high quality studies reported that age [29, 42, 48], gender [29, 62], prescribers’ length of practice [35, 85], graduating from a top-20 medical school [48, 62] did not influence prescribing decisions. A medium-quality study indicated general practitioners’ (GPs) who were a principal or partner in a practice were more likely to use new medicines than employee GPs [85].

#### Scope of expertise (*n* = 23)

Thirteen studies indicated that specialist prescribers adopted new medicines quicker than their other or primary care colleagues [22, 24, 28, 33, 41, 47, 48, 59, 61, 62, 73, 78] but only three were high-quality [24, 61, 78]. Other medium to high quality studies reported the opposite [29, 42, 48, 75] or no impact [35]. A high-quality study observed the clinical interest of primary care prescribers did not influence new medicine prescribing from the same clinical area [38]. Increasing total prescribing volume [48, 54, 85] or greater prescribing portfolio breadth [27, 54] in medium-quality studies, prescribing multiple medicines for the same condition [84] or larger prescription volume in the same therapeutic class [61] in high-quality studies were suggested to increase adoption of new medicines. Also, a high-quality study observed non-academic prescribers were more likely to use new and reformulated antipsychotics [23].

#### Knowledge and prescribing habits (*n* = 10)

Medium-quality studies suggested prescribers’ previous experience and knowledge of using new medicines increased their use [47, 67–69, 80, 86], whereas a lack of knowledge and confidence delayed or prevented use [50, 67]. Some prescribers commented that an overwhelming amount of new information for new medicines prescribing discouraged their use [67]. A high-quality study observed that continuing medical education activities supported prescribing of new medicine in one of two studied therapeutic areas [38]. In medium-quality studies, prescribers classed as early adopters in the past [27], or more likely to take clinical risks [85], or spend less time in patient consultations [85] tended to use new medicines quicker.

### Medicine-level factors

#### Efficacy (*n* = 6)

Some prescribers stated relative effectiveness of a new medicine influenced their prescribing decisions in medium-quality studies [50, 68, 69, 80, 86]. A high-quality study, focusing on novel chemotherapies, suggested that perceived better quality rather than incremental effectiveness influenced new medicine use [34].

#### Safety profile (*n* = 9)

Some prescribers reported that concerns over adverse effects [47, 50, 68, 80, 86] and unknown long-term risks [50] discouraged prescribing new medicines. Less interactions with other medicines or food [47, 68, 80], and less reported adverse effects [47] compared to existing treatments, encouraged uptake. All were medium-quality studies. Medium to high quality studies observed that national safety reports, e.g., Food and Drug Administration, highlighting safety concerns contributed to hesitancy of some prescribers to use new medicines [44, 83]. Also, a high-quality study suggested that scientific articles [32] rather than safety alerts influenced prescribing behaviours as safety concerns would be first reported in the scientific literature. Another medium-quality study suggested safety concerns with an existing class of medicines encouraged prescribers to use new medicines from a therapeutically different class [44].

#### Cost (n = 9)

Some prescribers reported a higher unit cost of a new medicine over existing therapy was a barrier for its use [47, 50, 68, 80, 86]. However, a proportion of prescribers did not consider a medicine’s cost in their prescribing decisions [47, 68, 86]. The unit cost of the new medicine was perceived differently by prescribers and patients. Patients appeared willing to pay more if the new medicine was in their best interest [80]. In contrast, prescribers considered the patient’s ability to pay out of pocket costs [22, 45, 47, 50, 71, 82], which could affect patients’ adherence to therapy and affordability of future prescriber’s visits [50]. Some prescribers also discussed their role in containing spending of social insurance, although others thought cost-savings to public spending was not a prescriber’s job [50]. Only two studies were high-quality [71, 82].

#### Therapeutic innovation (*n* = 5)

Two studies suggested new medicines [41] or reformulations [48], perceived as having therapeutic innovation, were adopted quicker than medicines without. Another study indicated the availability of more medicines within the same therapeutic category (i.e., higher competition) had a negative impact on new medicines entering the same category use [60]. Some prescribers reported considering a new medicine’s relative clinical benefits other than safety, efficacy, or cost over existing treatment [50, 80]. For instance, a positive effect on patient’s weight, comorbidities, and cardiovascular protection by new antidiabetic medicines [50]. All studies were medium quality.

#### Medicine administrative burden (n = 5)

Some prescribers stated the ease of administration [80] or use [50] of the medicine facilitated its uptake. Another study observed that increased complexity of taking a new medicine, e.g., twice a day, was a barrier to a minority of prescribers [47]. The majority of prescribers in the case of oral anticoagulants reported reduced monitoring or clinic visits encouraged their use [47, 68]. Also, concerns about difficulty to initiate new medicines negatively affected the willingness of some prescribers to use them, especially if they were less experienced [69]. All studies were medium quality.

### Organizational-level factors

#### Ownership status (*n* = 10)

Four high [37, 71, 81, 84] and three medium [57, 60, 65] quality studies suggested private, rather than public organizations, were more likely to use new medicines. Amongst private organizations, for-profit treatment programs were more likely to offer new medicines [37, 57]. In contrast, medium to high quality studies observed public organizations having greater use of new medicines [21, 46] or the ownership status did not influence the uptake [64].

#### Teaching status (*n* = 8)

Six medium studies [24, 63–65, 72, 73] and one high [77] quality study observed no difference in the uptake of new medicines between teaching and non-teaching hospitals. One medium-quality study, however, suggested a lower likelihood of new medicine use at a teaching hospital [74].

#### Size (*n* = 17)

Six high [35, 37, 46, 81, 82, 84] and five medium [48, 51, 57, 60, 68] quality studies indicated larger hospitals or practices were more likely to use new medicines. Other medium to high quality studies observed it for smaller [33, 75] or medium size [77] organizations. Also, three medium-quality studies suggested organization size did not influence the uptake [65, 72, 76].

#### Location (*n* = 16)

In some medium to high quality studies, organizations in urban areas [27, 57, 73], rural locations [42, 84], or in areas with fewer GPs [85] were observed to have a higher use of new medicines. Five medium to high quality studies reported geographical location having no impact on the uptake [27, 63, 71, 72, 77]. Also, nine studies reported regional variation in prescribing of new medicines [46, 60, 63, 65, 72, 74, 81, 85].

#### Available services and resources (*n* = 13)

In some cases, organizations providing, or having access to, related supportive services were more likely to adopt new medicines [37, 39, 57, 69, 76, 77, 79]; two were of high [69, 77] and one low [79] quality. For instance, detoxification, mental health services, or substance abuse counselling services for buprenorphine [37, 57, 69, 76] or stroke units for alteplase and direct oral anticoagulants [77, 79] were reported to facilitate the uptake. In other cases, supporting services such as the availability of heart failure clinics [35] or follow-up after hospitalisation [65] for sacubitril/valsartan, availability of hospital-based anticoagulant monitoring clinics for direct oral anticoagulants [53], or presence of dispensing services within general practices [27] had no impact. Also, prescribers reported lack of adequate time per patient visit acted as a barrier [67, 69], especially for less experienced prescribers [69]. Furthermore, some primary care clinicians suggested secondary care colleagues had more learning opportunities available (e.g., participation in clinical trials, education, and learning, access to more patients) supporting new medicine use [50].

#### Staff composition (*n* = 9)

Medium-quality studies indicated that lack of specialist prescribers was a barrier to new medicine use [21, 39, 48, 51, 57, 67]. For instance, organizations with more qualified staff [21] and GPs with hospital experience [59] were reported to adopt some of the studied medicines quicker. A high-quality study reported that organizations with higher numbers of nurses, and healthcare professionals with a generalist medical education, were more likely to use new medicines and the number of specialist prescribers had no influence [35]. Another medium-quality study reported the presence of clerical and nursing staff to have limited to no impact on the uptake [27].

#### Care co-ordination and quality (*n* = 3)

Some prescribers suggested that lack of organization and fragmentation in the provision of patient care [67], and non-clinical activities of care co-ordination, such as additional record-keeping requirements [69] were barriers to new medicine use. A study looking at heart failure treatment observed a lower uptake of a new medicine within hospitals scoring higher on non-heart failure service quality measures [65]. All studies were medium quality.

### External environment-level factors

#### Pharmaceutical detailing (*n* = 11)

The pharmaceutical industry was seen to promote awareness of new medicines through pharmaceutical detailing (pharmaceutical marketing aimed at prescribers) and indirectly through conferences, educational events, advertisements in academic and professional journals [50, 61, 80]. Prescribers in medium-quality studies had mixed views on its impact on their prescribing decisions [50, 68, 80] with some reporting pharmaceutical representatives as one of their main information sources about new medicines [50]. Three studies in USA indicated that current and/or past detailing with or without distribution of free samples had a positive impact on new medicine uptake [61, 66, 76]; two studies were high-quality [61, 66]. Also, organizations or areas with restricted access to pharmaceutical detailing or marketing regulation policies in place (e.g., ban of gifts, disclosure policy) had lower and slower uptake of new medicines [23, 33, 66], especially among primary care prescribers [33]; two studies were high-quality [23, 66]. A high quality study suggested gift restrictions having a greater negative impact than disclosure policies [66]. Another high-quality study indicated that prescribers completing training at medical schools with active policies restricting access to the pharmaceutical industry were less likely to use new medicines [55]. A medium-quality study suggested that prescribers with very low access to pharmaceutical detailing were slower in changing their prescribing behaviour when negative information about new medicines was released [33]. Lastly, a high-quality study reported direct-to-consumer advertising aimed at patients had no influence on the uptake [66].

#### Reimbursement conditions and formulary status (*n* = 13)

Nine studies suggested that reimbursement conditions for a medicine influenced the use of new medicines [23, 26, 29–31, 40, 50, 80, 83]; two high [29, 83] and one low [30] quality studies. Formulary or reimbursement restrictions [50, 80] or cost-control regulatory measures [23, 50] were suggested to have negative impact on new medicine use. Removing reimbursement restrictions such as a requirement of prior authorisation [29, 30], specialist use only in secondary care [40], only as second-line therapy [26, 83], or providing reimbursement for medicines excluded from a national formulary [31] were suggested to support new medicine use. The inclusion of new medicines in formularies (e.g., public insurance, regional, local, national) was reported to facilitate their use [37, 80] with one study being high-quality [37]. Also, medium to high quality studies suggested financial incentives to reduce prescribing costs had limited to no impact on the uptake of new medicines already included in formularies [27, 34].

#### Peer influence (internal and external) (*n* = 14)

Some prescribers indicated that their peers’ adoption of new medicines positively influenced their prescribing behaviour of new medicines in eight high [49, 52, 56, 61, 66, 71, 81, 84] and five medium [36, 50, 59, 68, 80] quality studies. Also, four high-quality studies suggested adoption of new medicines by prescribers after approval was even greater if their peers were early adopters [52, 71, 81, 84]. Four medium-quality studies suggested peers from secondary care or specialist areas influenced primary care prescribers [50, 59, 68, 80]. Some prescribers stated that other colleagues, opinion leaders, and experts influenced the use of new medicines [50, 59, 64, 80]. One high-quality study indicated peer influence being the greatest from month four of the medicine’s launch until month 17 [66]. Another high -quality study observed that peer influence had a greater impact in the states of the USA with policies restricting pharmaceutical marketing [56].

#### Guidelines (*n* = 6)

Guidelines (local, national, or international) were indicated to influence prescribing decisions of some prescribers [50, 68, 69, 86], especially of less experienced [50, 68, 69, 86]. Some prescribers reported absence of guidelines prevented [86] or delayed [50] prescribing new medicines till a guideline was released. In one study some prescribers suggested difficulties in determining the position for the new medicine within a clinical pathway was a barrier for the uptake [67]. Contrastingly, one study reported the publication of national guidelines had no impact on the rate of uptake of the studied new medicine [64]. All studies were medium quality.

#### Other information sources (*n* = 6)

Some prescribers in medium to high quality studies reported conferences, medical or news articles, scientific societies’ websites, or clinical trial reports discussing new medicines as having impact on prescribing decisions [32, 34, 50, 68, 76, 80]. A high-quality study, looking at cyclooxygenase-2 inhibitors, suggested that medical articles discouraged prescribers to use new medicines, but news articles and media reports encouraged it [32]. Another high-quality study, looking at oral chemotherapy agents, observed that clinical trials and media reports published around the Food and Drug Administration (in USA) approval date had positive impact on uptake [34]. Some prescribers reported scientific literature having greater influence in prescribing decisions than information gathered through social professional networks [50] or news media [34]. Also, prescribers using national research websites were suggested to use new medicines earlier [76].

#### Organization affiliations (n = 6)

Three studies indicated organizations’ participation in research networks having positive impact on new medicines use [21, 50, 57]. This was attributed to an organization’s experience with treatment protocols and exposure to the process of implementing new treatments. Also, organizational links with professional associations were reported to increase the likelihood of being an early adopter in the case of buprenorphine [76]. However, two studies suggested treatment programs affiliated with medical health centres had the same or slower adoption rates than the independent ones [39, 51]. All studies were medium quality.

## Discussion

### Summary of evidence

This systematic review has identified a broad range of factors affecting the uptake of new medicines within healthcare organizations. The identified factors were grouped into patient, prescriber, medicine, organizational, and external environment factors, as per the Chaudoir et al. [7] framework, and overlapped with the Consolidated Framework for Implementation Research (CFIR) [87]. They had a varied impact on the uptake of the different studied new medicines.

Our review findings, differently from earlier reviews [8–10], indicated the presence of patient influence on the uptake of new medicines. Patients were reported to influence prescribing decisions through their interest in or request for new medicines, satisfaction with current treatment, and therapy preferences. However, only a small number of studies reported patient influence and further research is required to establish its relative importance in the uptake of new medicines. Also, reviewed studies did not explore the impact of patient involvement in decision-making, availability of patient choice, and patient-clinician relationship, which are suggested to influence implementation of health innovations [7, 87, 88]. Understanding of these factors could offer explanation for why new medicines are used with some patient types but not others, as established in the reviewed studies.

In our review, high-quality studies indicated that patients with a higher education level were more likely to receive new medicines. This was in contrast to Lubloy’s review findings based on one study [10] and not reported in the other two earlier reviews [8, 9]. Patient education level has been associated with health education, literacy, and behaviours [89], potentially translating into the level of patient influence on new medicine use. Also, patient education level is linked with patient income [90]. As in Lubloy’s review [10], patients with higher income (able to pay out-of-pocket costs) and more comprehensive insurance plans were observed to have greater access to new medicines. This was more predominant in countries without universal health coverage, e.g., USA, but was also present in countries with universal health coverage requiring co-payments from patients, e.g., Taiwan. More studies are needed to explore the impact of patient income on new medicines use in countries with a publicly funded national health service, e.g. UK.

Another important finding was the impact of new medicine cost to healthcare organisations and patients on its uptake. Differently to previous reviews [8–10], our review findings indicated that the high cost of a new medicine was a barrier for the uptake, although to a varied extent. Increasing costs and expenditure on medicines and limited available funding to healthcare services is anticipated to influence uptake of high-cost new medicines [90]. Also, none of the reviewed studies considered the overall costs of new medicines compared to the established therapy (e.g., associated monitoring cost) or health economics (e.g., direct health costs), which could offer explanation to observed geographical variation and restrictions of new medicine use in the reviewed studies.

Our review findings indicated that formulary or reimbursement restrictions influence the uptake of new medicines, which was not reported in earlier reviews [8–10]. The purpose of formulary and reimbursement restrictions is to ensure evidence-based and cost-effective prescribing. These could be used as a cost-control measure for high-cost new medicines, limiting their use. Earlier reviews did not report on impact of guidelines [10] or concluded they had no [8] or varied impact [9]. Our review findings suggest that guidelines have an impact on the uptake. Inclusion of a new medicine in local or national guidelines establishes a new medicine’s place in existing clinical pathways and provides assurance to prescribers that they follow the best practice.

Lastly, the review findings reaffirmed that prescribers’ experience and knowledge, peer influence, pharmaceutical detailing, staff composition at organizations, and scientific literature influence uptake of new medicines [8–10]. However, the present review also highlighted that studies reporting factors affecting new medicine use lacked exploration of wider prescriber factors (e.g., motivation, values and goals, or beliefs about new medicines) and organizational factors (e.g., readiness for innovation, culture and climate, implementation process) reported in the implementation literature affecting implementation of health innovations [7, 87, 91]. Deficiency in reporting these factors could be due to the data sources used by the reviewed studies (mostly secondary administrative data from various databases) and a lack of theoretical frameworks used to inform study designs of reviewed studies. Only 20 of the reviewed studies referenced theoretical approaches employed but none of the studies addressed all constructs of the theoretical approach referenced. Future studies employing determinant frameworks or implementation theories [91] for primary data collection are required to address gaps in understanding barriers and facilitators to the implementation of new medicines into clinical practice.

### Strengths and limitations

This systematic review had a broad search strategy over seven databases and included studies of all methodological designs, conducted in both primary and secondary care settings. Grey literature and non-English language articles were excluded for pragmatic reasons and only studies published from 2008 and onwards were included, so other relevant studies might have been missed. The synthesis was underpinned by a determinant framework used in implementation science, which allowed the conceptualisation of the findings as provided in the review. Most of the reviewed studies were medium (38 studies) or high (26 studies) quality increasing confidence in the review findings. Finally, included studies covered medicines with varied complexities and expertise required to prescribe them. Therefore, not all influential factors identified in the review are relevant to all healthcare settings and medicines, reducing the generalisability of the review findings.

## Conclusions

This systematic review provides a comprehensive exploration of factors affecting the use of new medicines and identified potential gaps in the research literature, through the use of a determinant framework used in implementation science. Factors affecting new medicine use not reported in earlier reviews were identified and included the following: patient influence and education level, cost of new medicines, formulary and reimbursement restrictions, and guidelines. Further research employing determinant frameworks or implementation theories are needed to address identified gaps, especially regarding wider patient, prescriber, and organizational factors, in understanding barriers and facilitators to the uptake of new medicines into clinical practice.

## Supplementary Information

**Additional File 1.** PRISMA checklist.

**Additional File 2.** Medline Search Strategy.

**Additional File 3.** Methodological quality of included studies using the QATSDD tool.

**Additional File 4.** Summary of thematic areas identified in the included studies.

## Data Availability

All data generated or analysed during this study are included in this published article and additional files including articles included in the analysis which are cited in the reference list.

## Abbreviations

PRISMA: Preferred Reporting Items for Systematic Reviews and Meta-Analyses
QATSDD: Quality Assessment Tool for Studies with Diverse Designs
GP: General Practitioner
