# Supplementary material for: Barriers and facilitators to the uptake of new medicines into clinical practice: a systematic review
Source: BMC Health Serv Res. 2021 Nov 5;21:1198. doi: 10.1186/s12913-021-07196-4 (PMC8570007; doi:10.1186/s12913-021-07196-4)
Supplement: Supplementary file 2 — Additional File 2. Medline Search Strategy. [file 12913_2021_7196_MOESM2_ESM.docx]

Additional file 2: Medline Search Strategy

1. “uptake “
2. “implement*”
3. (MH “Drug Utilization” OR drug utili?ation OR MH “Drug Utilization Review”)
4. (MH “Diffusion of Innovation” OR diffusion of innovation)
5. “Sustainability”
6. S1 OR S2 OR S3 OR S4 OR S5)
7. “medicine*”
8. “drug*”
9. “treatment*”
10. “medical technolog*”
11. (MH “Pharmaceutical Preparations” OR pharmaceutical preparation*)
12. “medical therapy*”
13. S7 OR S8 OR S9 OR S10 OR S11 OR S12)
14. “new”
15. “innovat*”
16. “novel”
17. “advance*”
18. “inventive”
19. S14 OR S15 OR S16 OR S17 OR S18
20. (MH “Primary Health Care” OR primary health care)
21. (MH “Secondary Care” OR MH “Secondary Care Centers” OR secondary care)
22. “nhs OR national health service”
23. “clinical commissioning group*”
24. (MH “General Practice” OR general practitioner practice)
25. (MH “Family Practice” OR family practice)
26. (MH “Group Practice” OR group practice OR MH “Hospitals, Group Practice”)
27. (MH “Health Services” OR health service*)
28. “hospital* “
29. (MH “Hospitals”)
30. S20 OR S21 OR S22 OR S23 OR S24 OR S25 OR S26 OR S27 OR S28 OR S29
31. “Barrier*”
32. “obstacle*”
33. “imped*”
34. “challenge*”
35. “limit*”
36. “hinder*”
37. “prevent*”
38. “inhibit*”
39. “restrict*”
40. “enabler*”
41. “facillitat*”
42. “access*”
43. “promot*”
44. “encourage*”
45. “stimulat*”
46. S31 OR S32 OR S33 OR S34 OR S35 OR S36 OR S37 OR S38 OR S39 OR S40 OR S41 OR S42 OR S43 ORS#44 OR S45
47. S6 AND S13 AND S19 AND S30 AND S46
48. S47 limit to English language
49. S48 limit to Date of Publication: 2008/01/01-2020/04/23
