# Supplementary material for: Barriers and facilitators to the uptake of new medicines into clinical practice: a systematic review
Source: BMC Health Serv Res. 2021 Nov 5;21:1198. doi: 10.1186/s12913-021-07196-4 (PMC8570007; doi:10.1186/s12913-021-07196-4)
Supplement: Supplementary file 3 — Additional File 3. Methodological quality of included studies using the QATSDD tool. [file 12913_2021_7196_MOESM3_ESM.docx]

# Additional File 3: Table S:1. Methodological quality of included studies using the QASTDD tool. Colour coding: green- high quality (≥70.0%), yellow- medium quality (50% to <70.0%), red- low quality (<50%) studies.

| QASTDD tool criteria | | Abraham et al. (2010) [78] | | AbuDagga et al. (2014) [39] | | Anderson et al. (2015) [67] | | Anderson et al. (2018) [70] | | Baik et al. (2016) [51] | Boon et al. (2008) [82] | | Bourke et al. (2012) [22] | | Brais et al. (2017) [40] | |  |
| --- | --- | --- | --- | --- | --- | --- | --- | --- | --- | --- | --- | --- | --- | --- | --- | --- | --- |
| 1. Explicit theoretical framework | | 3 | | 1 | | 1 | | 1 | | 1 | 2 | | 3 | | 1 | |  |
| 1. Statement of aims/objectives in main body of report | | 3 | | 3 | | 2 | | 3 | | 3 | 3 | | 2 | | 3 | |  |
| 1. Clear description of research setting | | 3 | | 3 | | 3 | | 3 | | 2 | 3 | | 3 | | 3 | |  |
| 1. Evidence of sample size considered in terms of analysis | | 2 | | 1 | | 2 | | 1 | | 1 | 2 | | 2 | | 1 | |  |
| 1. Representative sample of target group of a reasonable size | | 3 | | 2 | | 3 | | 2 | | 2 | 2 | | 2 | | 2 | |  |
| 1. Description of procedure for data collection | | 3 | | 3 | | 2 | | 1 | | 3 | 3 | | 2 | | 3 | |  |
| 1. Rationale for choice of data collection tool(s) | | 0 | | 2 | | 3 | | 2 | | 0 | 2 | | 2 | | 1 | |  |
| 1. Detailed recruitment data | | 3 | | 1 | | 3 | | 3 | | 3 | 2 | | 1 | | 3 | |  |
| 1. QUANTITATIVE only: Statistical Assessment of reliability and validity of measurement tool(s) | | 0 | | 0 | | 2 | | 3 | | 0 | 0 | | 1 | | 1 | |  |
| 1. QUANTITATIVE only: Fit between stated research question and method of data collection | | 2 | | 3 | | 3 | | 3 | | 3 | 2 | | 3 | | 3 | |  |
| 1. QUALITATIVE only: Fit between stated research question and format and content of data collection tool e.g. interview schedule | |  | |  | |  | |  | |  |  | |  | |  | |  |
| 1. Fit between research question and method analysis | | 3 | | 3 | | 3 | | 3 | | 3 | 3 | | 3 | | 3 | |  |
| 1. Good justification for analytical method selected | | 1 | | 2 | | 3 | | 3 | | 3 | 2 | | 3 | | 2 | |  |
| 1. QUALITATIVE only: Assessment of reliability of analytical process | |  | |  | |  | |  | |  |  | |  | |  | |  |
| 1. Evidence of user involvement in design | | 0 | | 0 | | 0 | | 0 | | 0 | 0 | | 0 | | 0 | |  |
| 1. Strengths and limitations critically discussed | | 2 | | 2 | | 2 | | 2 | | 3 | 2 | | 2 | | 2 | |  |
| Total score/maximum score | | 28/42 | | 26/42 | | 32/42 | | 30/42 | | 27/42 | 28/42 | | 29/42 | | 28/42 | |  |
| Total score (%) | | 67% | | 62% | | 76% | | 71% | | 64% | 67% | | 69% | | 67% | |  |
| QASTDD tool criteria | Burden et al. (2015) [56] | | Carracedo-Martinez et al al. (2017) [83] | | Chamberlain et al. (2014) [84] | | Chitagunta et al. (2009) [52] | | Chressanthis et al. (2012) [35] | | | Conti et al. (2012) [64] | | DeVore et al. (2018) [41] | | Donohue et al. (2018) [86] | |
| 1. Explicit theoretical framework | 2 | | 1 | | 1 | | 2 | | 2 | | | 3 | | 2 | | 2 | |
| 1. Statement of aims/objectives in main body of report | 3 | | 2 | | 3 | | 2 | | 3 | | | 3 | | 3 | | 3 | |
| 1. Clear description of research setting | 3 | | 2 | | 3 | | 3 | | 2 | | | 3 | | 2 | | 3 | |
| 1. Evidence of sample size considered in terms of analysis | 2 | | 1 | | 2 | | 2 | | 2 | | | 2 | | 3 | | 1 | |
| 1. Representative sample of target group of a reasonable size | 3 | | 1 | | 3 | | 2 | | 2 | | | 2 | | 3 | | 2 | |
| 1. Description of procedure for data collection | 3 | | 2 | | 3 | | 3 | | 2 | | | 3 | | 3 | | 2 | |
| 1. Rationale for choice of data collection tool(s) | 1 | | 2 | | 2 | | 2 | | 2 | | | 1 | | 2 | | 1 | |
| 1. Detailed recruitment data | 3 | | 0 | | 3 | | 2 | | 1 | | | 1 | | 0 | | 3 | |
| 1. QUANTITATIVE only: Statistical Assessment of reliability and validity of measurement tool(s) | 1 | | 1 | | 0 | | 2 | | 1 | | | 2 | | 0 | | 0 | |
| 1. QUANTITATIVE only: Fit between stated research question and method of data collection | 3 | | 3 | | 3 | | 3 | | 2 | | | 2 | | 3 | | 3 | |
| 1. QUALITATIVE only: Fit between stated research question and format and content of data collection tool e.g. interview schedule |  | |  | |  | |  | |  | | |  | |  | |  | |
| 1. Fit between research question and method analysis | 3 | | 3 | | 3 | | 3 | | 3 | | | 3 | | 3 | | 3 | |
| 1. Good justification for analytical method selected | 2 | | 0 | | 3 | | 2 | | 3 | | | 3 | | 3 | | 3 | |
| 1. QUALITATIVE only: Assessment of reliability of analytical process |  | |  | |  | |  | |  | | |  | |  | |  | |
| 1. Evidence of user involvement in design | 0 | | 0 | | 0 | | 0 | | 0 | | | 0 | | 0 | | 0 | |
| 1. Strengths and limitations critically discussed | 2 | | 1 | | 2 | | 2 | | 1 | | | 2 | | 3 | | 3 | |
| Total score/maximum score | 31/42 | | 19/42 | | 31/42 | | 30/42 | | 26/42 | | | 30/42 | | 31/42 | | 29/42 | |
| Total score (%) | 74% | | 45% | | 74% | | 71% | | 62% | | | 71% | | 74% | | 69% | |

| QASTDD tool criteria | | Ducharme and Abraham (2008) [74] | | Dybdhal et al. (2011) [23] | Friedman et al. (2010) [81] | | Fuksa et al. (2015) [85] | Garjon et al. (2012) [24] | | Groves et al. (2010) [25] | | Haider et al. (2008) [58] | | Hickson et al. (2019) [73] | Hirundassamee and Ratanawijitrasin (2009) [60] | | | Hsieh and Liu (2012) [65] | |
| --- | --- | --- | --- | --- | --- | --- | --- | --- | --- | --- | --- | --- | --- | --- | --- | --- | --- | --- | --- |
| 1. Explicit theoretical framework | | 3 | | 2 | 3 | | 1 | 1 | | 3 | | 1 | | 2 | 1 | | | 1 | |
| 1. Statement of aims/objectives in main body of report | | 3 | | 3 | 3 | | 3 | 3 | | 3 | | 3 | | 3 | 3 | | | 3 | |
| 1. Clear description of research setting | | 3 | | 3 | 2 | | 3 | 3 | | 3 | | 3 | | 2 | 3 | | | 3 | |
| 1. Evidence of sample size considered in terms of analysis | | 1 | | 2 | 2 | | 1 | 2 | | 2 | | 1 | | 2 | 1 | | | 1 | |
| 1. Representative sample of target group of a reasonable size | | 3 | | 2 | 2 | | 2 | 2 | | 3 | | 3 | | 3 | 2 | | | 3 | |
| 1. Description of procedure for data collection | | 3 | | 3 | 3 | | 3 | 2 | | 3 | | 3 | | 2 | 3 | | | 3 | |
| 1. Rationale for choice of data collection tool(s) | | 2 | | 2 | 1 | | 1 | 2 | | 3 | | 2 | | 1 | 1 | | | 3 | |
| 1. Detailed recruitment data | | 3 | | 3 | 2 | | 1 | 3 | | 3 | | 2 | | 3 | 2 | | | 2 | |
| 1. QUANTITATIVE only: Statistical Assessment of reliability and validity of measurement tool(s) | | 0 | | 0 | 1 | | 0 | 0 | | 0 | | 0 | | 0 | 0 | | | 1 | |
| 1. QUANTITATIVE only: Fit between stated research question and method of data collection | | 3 | | 3 | 2 | | 3 | 3 | | 2 | | 3 | | 3 | 3 | | | 3 | |
| 1. QUALITATIVE only: Fit between stated research question and format and content of data collection tool e.g. interview schedule | |  | |  |  | |  |  | |  | |  | |  |  | | |  | |
| 1. Fit between research question and method analysis | | 3 | | 3 | 3 | | 3 | 2 | | 3 | | 3 | | 3 | 2 | | | 3 | |
| 1. Good justification for analytical method selected | | 3 | | 3 | 1 | | 2 | 1 | | 3 | | 3 | | 3 | 1 | | | 3 | |
| 1. QUALITATIVE only: Assessment of reliability of analytical process | |  | |  |  | |  |  | |  | |  | |  |  | | |  | |
| 1. Evidence of user involvement in design | | 0 | | 0 | 0 | | 0 | 0 | | 0 | | 0 | | 0 | 0 | | | 0 | |
| 1. Strengths and limitations critically discussed | | 2 | | 1 | 2 | | 2 | 1 | | 2 | | 3 | | 2 | 0 | | | 2 | |
| Total score/maximum score | | 32/42 | | 30/42 | 27/42 | | 25/42 | 25/42 | | 33/42 | | 30/42 | | 29/42 | 22/42 | | | 31/42 | |
| Total score (%) | | 76% | | 71% | 64% | | 60% | 60% | | 79% | | 71% | | 69% | 52% | | | 74% | |
| QASTDD tool criteria | | Huang et al. (2013) [61] | | Huskamp et al. (2013) [26] | | Iyengar et al. (2011) [27] | Karampli et al. (2020) [59] | | | Keating et al. (2018) [42] | | Keating et al. (2020) [43] | | Kennedy et al. (2020) [66] | | | Kerezsturi et al. (2015) [68] | King et al. (2013) [38] | |
| 1. Explicit theoretical framework | | 1 | | 1 | | 3 | 3 | | | 1 | | 3 | | 2 | | | 1 | 2 | |
| 1. Statement of aims/objectives in main body of report | | 3 | | 3 | | 3 | 3 | | | 3 | | 3 | | 3 | | | 2 | 3 | |
| 1. Clear description of research setting | | 3 | | 3 | | 3 | 2 | | | 2 | | 2 | | 2 | | | 3 | 2 | |
| 1. Evidence of sample size considered in terms of analysis | | 1 | | 1 | | 1 | 3 | | | 1 | | 1 | | 1 | | | 1 | 2 | |
| 1. Representative sample of target group of a reasonable size | | 2 | | 2 | | 2 | 2 | | | 2 | | 2 | | 2 | | | 2 | 3 | |
| 1. Description of procedure for data collection | | 2 | | 2 | | 3 | 2 | | | 3 | | 3 | | 2 | | | 2 | 3 | |
| 1. Rationale for choice of data collection tool(s) | | 0 | | 3 | | 3 | 0 | | | 0 | | 2 | | 2 | | | 1 | 3 | |
| 1. Detailed recruitment data | | 2 | | 2 | | 2 | 0 | | | 0 | | 3 | | 3 | | | 1 | 2 | |
| 1. QUANTITATIVE only: Statistical Assessment of reliability and validity of measurement tool(s) | | 0 | | 0 | | 3 |  | | | 3 | | 3 | | 0 | | | 3 | 1 | |
| 1. QUANTITATIVE only: Fit between stated research question and method of data collection | | 2 | | 3 | | 3 |  | | | 3 | | 3 | | 3 | | | 3 | 3 | |
| 1. QUALITATIVE only: Fit between stated research question and format and content of data collection tool e.g. interview schedule | |  | |  | |  | 2 | | |  | |  | |  | | |  |  | |
| 1. Fit between research question and method analysis | | 3 | | 3 | | 3 | 3 | | | 3 | | 3 | | 3 | | | 3 | 3 | |
| 1. Good justification for analytical method selected | | 3 | | 3 | | 3 | 2 | | | 3 | | 3 | | 3 | | | 3 | 3 | |
| 1. QUALITATIVE only: Assessment of reliability of analytical process | |  | |  | |  | 1 | | |  | |  | |  | | |  |  | |
| 1. Evidence of user involvement in design | | 0 | | 0 | | 0 | 0 | | | 0 | | 0 | | 0 | | | 0 | 0 | |
| 1. Strengths and limitations critically discussed | | 2 | | 2 | | 2 | 3 | | | 2 | | 2 | | 3 | | | 2 | 2 | |
| Total score/maximum score | | 26/42 | | 28/42 | | 34/42 | 26/42 | | | 26/42 | | 31/42 | | 29/42 | | | 27/42 | 32/42 | |
| Total score (%) | | 62% | | 67% | | 81% | 62% | | | 62% | | 74% | | 69% | | | 64% | 76% | |

| QASTDD tool criteria | King and Bearman (2017) [87] | Knudsen et al. (2009) [75] | Lin H et al. (2011) [44] | Lin S et al. (2011) [28] | Liu et al. (2011) [29] | Liu and Gupta (2011) [30] | Lo-Ciganic et al. (2016) [63] | Luo et al. (2017) [45] | Luo et al. (2018) [78] | Luo et al. (2019) [76] |
| --- | --- | --- | --- | --- | --- | --- | --- | --- | --- | --- |
| 1. Explicit theoretical framework | 2 | 3 | 3 | 3 | 3 | 3 | 1 | 0 | 2 | 2 |
| 1. Statement of aims/objectives in main body of report | 2 | 3 | 3 | 2 | 3 | 3 | 2 | 2 | 3 | 3 |
| 1. Clear description of research setting | 3 | 3 | 2 | 3 | 2 | 3 | 3 | 2 | 2 | 2 |
| 1. Evidence of sample size considered in terms of analysis | 1 | 1 | 1 | 2 | 2 | 2 | 1 | 1 | 1 | 1 |
| 1. Representative sample of target group of a reasonable size | 3 | 2 | 2 | 2 | 2 | 3 | 2 | 2 | 2 | 2 |
| 1. Description of procedure for data collection | 3 | 2 | 2 | 1 | 2 | 1 | 2 | 3 | 3 | 1 |
| 1. Rationale for choice of data collection tool(s) | 3 | 1 | 2 | 3 | 2 | 1 | 3 | 1 | 1 | 2 |
| 1. Detailed recruitment data | 2 | 3 | 1 | 2 | 1 | 0 | 2 | 0 | 0 | 3 |
| 1. QUANTITATIVE only: Statistical Assessment of reliability and validity of measurement tool(s) | 3 | 0 | 0 | 1 | 0 | 3 | 0 | 0 | 3 | 1 |
| 1. QUANTITATIVE only: Fit between stated research question and method of data collection | 3 | 2 | 3 | 2 | 3 | 3 | 3 | 3 | 3 | 3 |
| 1. QUALITATIVE only: Fit between stated research question and format and content of data collection tool e.g. interview schedule |  |  |  |  |  |  |  |  |  |  |
| 1. Fit between research question and method analysis | 3 | 3 | 3 | 3 | 3 | 3 | 3 | 3 | 3 | 3 |
| 1. Good justification for analytical method selected | 3 | 2 | 3 | 3 | 3 | 3 | 3 | 2 | 3 | 3 |
| 1. QUALITATIVE only: Assessment of reliability of analytical process |  |  |  |  |  |  |  |  |  |  |
| 1. Evidence of user involvement in design | 0 | 0 | 0 | 0 | 0 | 0 | 0 | 0 | 0 | 0 |
| 1. Strengths and limitations critically discussed | 2 | 2 | 2 | 2 | 1 | 2 | 3 | 2 | 2 | 2 |
| Total score/maximum score | 33/42 | 27/42 | 27/42 | 29/42 | 27/42 | 30/42 | 28/42 | 21/42 | 28/42 | 28/42 |
| Total score (%) | 79% | 64% | 64% | 69% | 64% | 71% | 67% | 50% | 67% | 67% |

| QASTDD tool criteria | Manchanda et al. (2008) [31] | Martin et al. (2017) [71] | Murphy et al. (2018) [57] | Netherland et al. (2009) [70] | Ohl et al. (2013) [53] | Ohlsson et al. (2009) [32] | Patel et al. (2015) [46] | Potpara et al. (2017) [55] | Rodwin et al. (2020) [47] | Sato et al. (2012) [36] |
| --- | --- | --- | --- | --- | --- | --- | --- | --- | --- | --- |
| 1. Explicit theoretical framework | 3 | 1 | 1 | 3 | 2 | 3 | 1 | 2 | 2 | 1 |
| 1. Statement of aims/objectives in main body of report | 3 | 3 | 3 | 3 | 2 | 3 | 3 | 2 | 3 | 3 |
| 1. Clear description of research setting | 3 | 3 | 3 | 2 | 3 | 3 | 2 | 3 | 2 | 3 |
| 1. Evidence of sample size considered in terms of analysis | 2 | 3 | 1 | 1 | 2 | 2 | 1 | 1 | 1 | 1 |
| 1. Representative sample of target group of a reasonable size | 2 | 2 | 1 | 2 | 2 | 2 | 2 | 2 | 2 | 2 |
| 1. Description of procedure for data collection | 2 | 3 | 1 | 2 | 3 | 3 | 2 | 3 | 2 | 2 |
| 1. Rationale for choice of data collection tool(s) | 2 | 2 | 1 | 1 | 2 | 3 | 1 | 2 | 1 | 2 |
| 1. Detailed recruitment data | 2 | 3 | 3 | 1 | 3 | 1 | 3 | 3 | 3 | 1 |
| 1. QUANTITATIVE only: Statistical Assessment of reliability and validity of measurement tool(s) | 3 |  | 1 | 0 | 3 | 0 | 1 | 0 | 1 | 0 |
| 1. QUANTITATIVE only: Fit between stated research question and method of data collection | 3 |  | 2 | 2 | 3 | 3 | 3 | 2 | 3 | 3 |
| 1. QUALITATIVE only: Fit between stated research question and format and content of data collection tool e.g. interview schedule | 2 | 3 |  |  |  |  |  |  |  |  |
| 1. Fit between research question and method analysis | 3 | 3 | 2 | 3 | 3 | 3 | 3 | 3 | 3 | 3 |
| 1. Good justification for analytical method selected | 3 | 1 | 1 | 2 | 3 | 3 | 3 | 2 | 3 | 3 |
| 1. QUALITATIVE only: Assessment of reliability of analytical process | 0 | 1 |  |  |  |  |  |  |  |  |
| 1. Evidence of user involvement in design | 0 | 0 | 0 | 0 | 0 | 0 | 0 | 0 | 0 | 0 |
| 1. Strengths and limitations critically discussed | 3 | 1 | 1 | 2 | 3 | 1 | 2 | 1 | 2 | 0 |
| Total score/maximum score | 36/48 | 29/42 | 21/42 | 24/42 | 34/42 | 30/42 | 27/42 | 26/42 | 28/42 | 24/42 |
| Total score (%) | 75% | 69% | 50% | 57% | 81% | 71% | 64% | 62% | 67% | 57% |

| QASTDD tool criteria | Savage et al. (2012) [80] | Scholten et al. (2015) [79] | Steinberg et al. (2013) [48] | Tanislav et al. (2018) [49] | Tobin et al. (2008) [62] | Tsai et al. (2010) [33] | Wang et al. (2010) [50] | Weir et al. (2012) [37] | Wen et al. (2011) [34] | Zhang et al. (2019) [54] | Zhang et al. (2020) [72] |
| --- | --- | --- | --- | --- | --- | --- | --- | --- | --- | --- | --- |
| 1. Explicit theoretical framework | 3 | 3 | 0 | 0 | 1 | 2 | 2 | 1 | 3 | 3 | 1 |
| 1. Statement of aims/objectives in main body of report | 3 | 3 | 3 | 2 | 3 | 3 | 3 | 3 | 3 | 2 | 2 |
| 1. Clear description of research setting | 2 | 3 | 3 | 3 | 3 | 3 | 2 | 3 | 3 | 3 | 3 |
| 1. Evidence of sample size considered in terms of analysis | 1 | 1 | 3 | 1 | 0 | 2 | 2 | 2 | 3 | 1 | 1 |
| 1. Representative sample of target group of a reasonable size | 2 | 2 | 3 | 2 | 2 | 3 | 2 | 3 | 3 | 2 | 2 |
| 1. Description of procedure for data collection | 1 | 3 | 3 | 1 | 3 | 2 | 3 | 3 | 2 | 3 | 2 |
| 1. Rationale for choice of data collection tool(s) | 2 | 3 | 3 | 2 | 0 | 2 | 2 | 2 | 1 | 2 | 2 |
| 1. Detailed recruitment data | 2 | 2 | 3 | 1 | 2 | 3 | 3 | 2 | 3 | 2 | 2 |
| 1. QUANTITATIVE only: Statistical Assessment of reliability and validity of measurement tool(s) | 1 | 0 | 3 | 0 |  | 0 | 0 | 1 | 0 | 0 | 0 |
| 1. QUANTITATIVE only: Fit between stated research question and method of data collection | 3 | 3 | 3 | 3 |  | 2 | 3 | 3 | 2 | 3 | 3 |
| 1. QUALITATIVE only: Fit between stated research question and format and content of data collection tool e.g. interview schedule |  |  |  |  | 2 |  |  |  |  |  |  |
| 1. Fit between research question and method analysis | 3 | 3 | 3 | 3 | 2 | 3 | 3 | 3 | 3 | 3 | 2 |
| 1. Good justification for analytical method selected | 3 | 2 | 1 | 1 | 1 | 3 | 3 | 3 | 3 | 2 | 0 |
| 1. QUALITATIVE only: Assessment of reliability of analytical process |  |  |  |  | 2 |  |  |  |  |  |  |
| 1. Evidence of user involvement in design | 0 | 0 | 0 | 0 | 0 | 0 | 0 | 0 | 0 | 0 | 0 |
| 1. Strengths and limitations critically discussed | 2 | 2 | 2 | 0 | 0 | 2 | 2 | 2 | 2 | 3 | 1 |
| Total score/maximum score | 28/42 | 30/42 | 33/42 | 19/42 | 21/42 | 30/42 | 30/42 | 31/42 | 31/42 | 29/42 | 21/42 |
| Total score (%) | 67% | 71% | 79% | 45% | 50% | 71% | 71% | 74% | 74% | 69% | 50% |
