# Supplementary material for: Barriers and facilitators to the uptake of new medicines into clinical practice: a systematic review
Source: BMC Health Serv Res. 2021 Nov 5;21:1198. doi: 10.1186/s12913-021-07196-4 (PMC8570007; doi:10.1186/s12913-021-07196-4)
Supplement: Supplementary file 4 — Additional File 4. Summary of thematic areas identified in the included studies. [file 12913_2021_7196_MOESM4_ESM.docx]

Additional file 4: Summary of thematic areas identified in the included studies.

|  | **Prescriber factors** | **Patient factors** | **Medicine factors** | **Organizational factors** | **External environment factors** |
| --- | --- | --- | --- | --- | --- |
| Abraham et al (2010) [77] |  |  |  | √ | √ |
| AbuDagga et al. (2014) [39] | √ | √ | √ |  |  |
| Anderson et al. (2015) [67] | √ |  |  |  | √ |
| Anderson et al (2018) [69] | √ |  |  |  |  |
| Baik et al. (2016) [51] |  | √ |  |  |  |
| Boon et al. (2008) [82] |  |  |  |  | √ |
| Bourke and Roper (2012) [22] | √ |  |  | √ | √ |
| Brais et al. (2017) [40] | √ | √ |  |  |  |
| Burden et al. (2015) [56] | √ | √ |  |  | √ |
| Carracedo-Martínez et al. (2017) [83] |  |  |  |  | √ |
| Chamberlain et al. (2014) [84] |  |  |  |  | √ |
| Chitagunta et al. (2009) [52] |  | √ | √ |  | √ |
| Chressanthis et al. (2012) [35] | √ |  |  | √ | √ |
| Conti et al. (2012) [64] |  | √ | √ |  | √ |
| DeVore et al. (2018) [41] | √ | √ |  | √ |  |
| Donohue et al. (2018) [86] |  |  |  |  | √ |
| Ducharme and Abraham (2008) [74] |  |  |  | √ | √ |
| Dybdahl et al. (2011) [23] | √ |  |  |  |  |
| Friedman et al. (2010) [81] |  |  |  | √ | √ |
| Fuksa et al. (2015) [85] |  |  |  |  | √ |
| Garjon et al. (2012) [24] | √ |  | √ |  |  |
| Groves et al. (2010) [25] | √ |  |  | √ |  |
| Haider et al. (2008) [58] |  | √ |  |  |  |
| Hickson et al. (2019) [73] |  |  | √ |  | √ |
| Hirunrassamee and Ratanawijitrasin (2009) [60] |  | √ | √ |  |  |
| Hsieh and Liu (2012) [65] |  | √ |  | √ |  |
| Huang et al. (2013) [61] | √ | √ | √ |  |  |
|  | **Prescriber factors** | **Patient factors** | **Medicine factors** | **Organisational factors** | **External environment factors** |
| Huskamp et al (2013) [26] | √ |  | √ | √ |  |
| Iyengar et al. (2011) [27] |  |  |  |  | √ |
| Karampli et al. (2020) [59] | √ | √ | √ | √ | √ |
| Keating et al. (2018) [42] |  | √ |  | √ | √ |
| Keating et al. (2020) [43] | √ | √ |  |  | √ |
| Kennedy et al. (2020) [66] |  |  |  | √ |  |
| Kereszturi et al. (2015) [68] | √ |  |  | √ |  |
| King et al. (2013) [38] |  |  |  |  | √ |
| King and Bearman (2017) [87] |  |  |  |  | √ |
| Knudsen et al. (2009) [75] |  |  |  | √ | √ |
| Lin H et al. (2011) [44] |  | √ |  |  |  |
| Lin S et al. (2011) [28] | √ |  |  |  | √ |
| Liu et al. (2011) [29] |  | √ | √ | √ |  |
| Liu and Gupta (2012) [30] | √ | √ |  |  | √ |
| Lo-Ciganic et al. (2016) [63] | √ | √ |  | √ |  |
| Luo et al. (2017) [45] |  | √ |  | √ |  |
| Luo et al. (2018) [78] |  |  |  | √ | √ |
| Luo et al. (2019) [76] |  |  |  | √ |  |
| Machanda et al. (2008) [31] |  |  |  |  | √ |
| Martin et al. (2017) [71] | √ |  |  | √ | √ |
| Murphy et al. (2018) [57] | √ | √ | √ | √ | √ |
| Netherland et al. (2009) [70] | √ | √ | √ | √ | √ |
| Ohl et al. (2013) [53] |  | √ |  |  |  |
| Ohlsson et al. (2009) [32] |  | √ | √ | √ | √ |
| Patel et al. (2015) [46] |  | √ |  | √ |  |
| Potpara et al. (2017) [55] | √ | √ |  | √ |  |
| Rodwin et al. (2020) [47] |  | √ |  | √ |  |
| Sato et al. (2012) [36] | √ |  | √ | √ |  |
| Savage et al. (2012) [80] |  |  |  | √ | √ |
| Scholten et al. (2015) [79] |  |  |  | √ |  |
| Steinberg et al. (2013) [48] | √ | √ |  |  |  |
| Tanislav et al (2018) [49] |  | √ |  | √ |  |
|  | **Prescriber factors** | **Patient factors** | **Medicine factors** | **Organisational factors** | **External environment factors** |
| Tobin et al (2008) [62] | √ | √ | √ |  | √ |
| Tsai et al (2010) [33] |  |  |  | √ | √ |
| Wang et al. (2010) [50] |  | √ | √ | √ |  |
| Weir et al. (2012) [37] |  |  | √ |  | √ |
| Wen et al. (2011) [34] | √ |  |  | √ | √ |
| Zhang et al. (2019) [54] | √ | √ |  | √ |  |
| Zhang et al. (2020) [72] | √ |  | √ | √ | √ |
